# Supplementary material for: Restaining-based annotation for cancer histology segmentation to overcome annotation-related limitations among pathologists
Source: Patterns (N Y). 2023 Feb 10;4(2):100688. doi: 10.1016/j.patter.2023.100688 (PMC9982301; doi:10.1016/j.patter.2023.100688)
Supplement: Document S1. Figures S1–S15 and Tables S1, S4, and S6 [file mmc1.pdf]

Patterns, Volume *4*

Supplemental information

Restaining-based annotation for cancer histology segmentation to overcome annotation-related limitations among pathologists

Daisuke Komura, Takumi Onoyama, Koki Shinbo, Hiroto Odaka, Minako Hayakawa, Mieko Ochi, Ranny Rahaningrum Herdiantoputri, Haruya Endo, Hiroto Katoh, Tohru Ikeda, Tetsuo Ushiku, and Shumpei Ishikawa

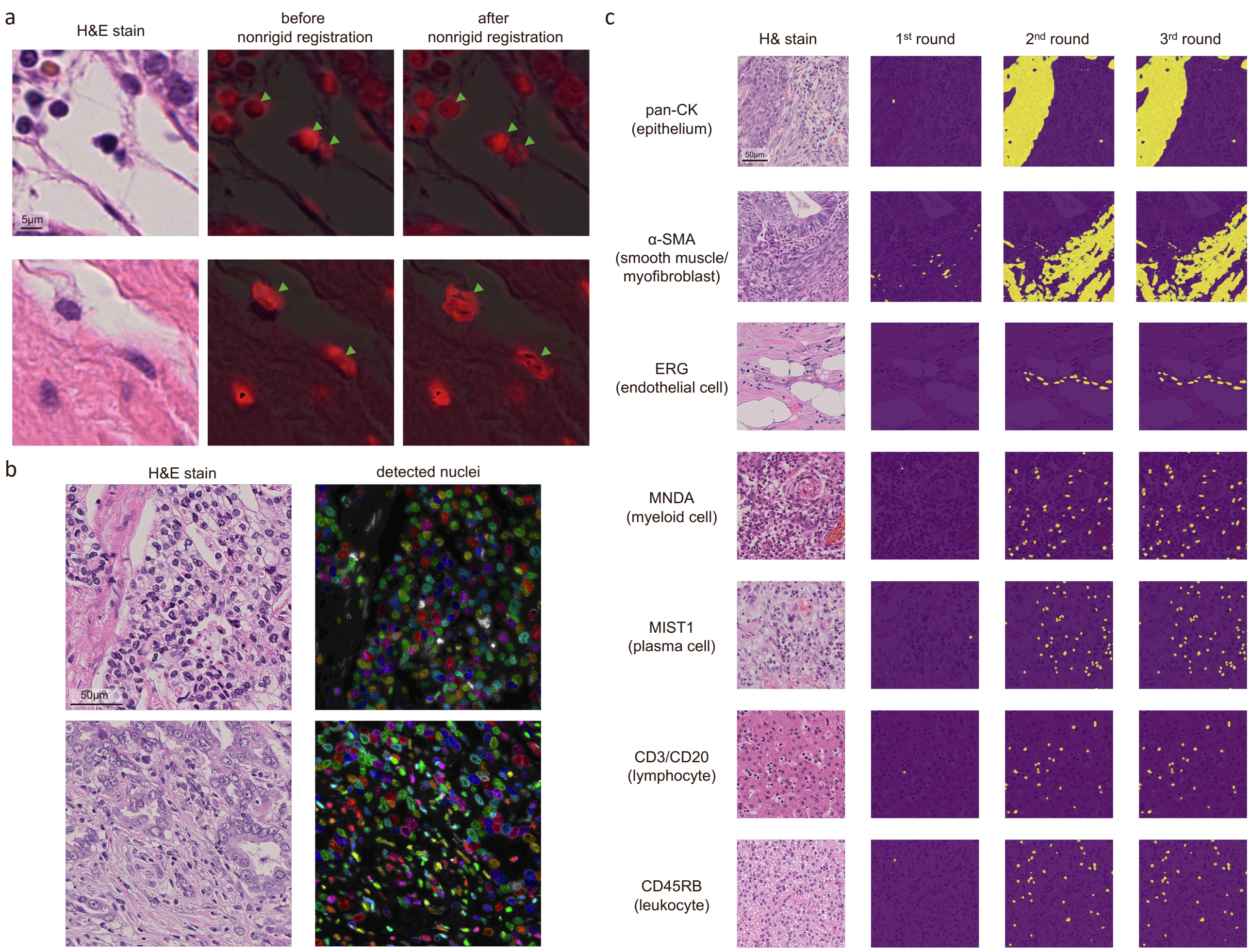

Figure S1. Preprocessing of H&E and the IF slides, related to Figure 1. a, H&E-stained image, along with DAPI channel images before and after nonrigid registration between estimated haematoxylin components in H&E-stained image. H&E images are overlayed in the DAPI channel images. The DAPI component is shown in red, which is converted from blue to improve visibility, in the middle and right images. Arrowheads indicate cells drastically corrected by nonrigid registration. **b,** H&E-stained image and detected nuclei in DAPI component in IF slides. Colour regions represent detected nuclei and grey regions represent nuclei candidates that did not reach the detection threshold. **c,** IF threshold improvement in each phase. The scales of all the image patches in each panel are the same.

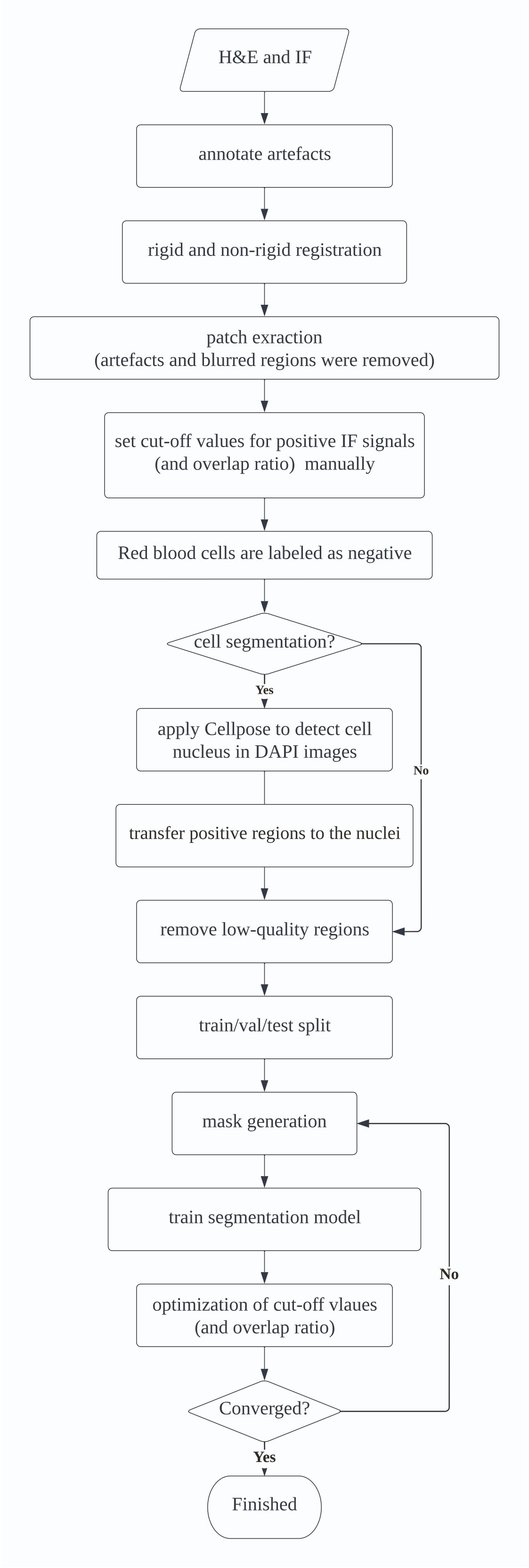

Figure S2. Flow chart of SegPath generation, related to Figure 1.

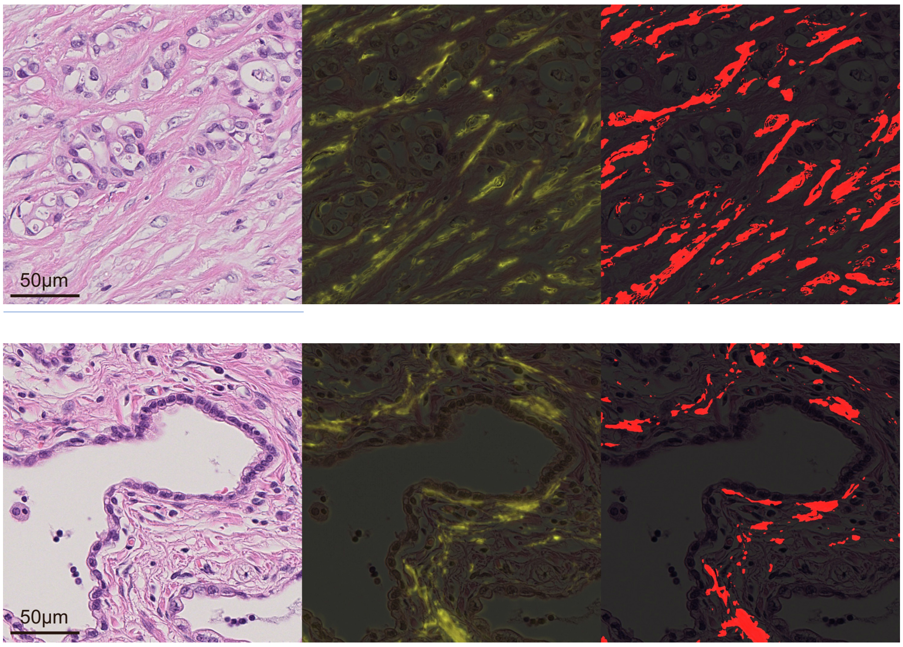

Figure S3. Weak staining of αSMA, related to Figure 3. Each triplet shows an H&E-stained image, the corresponding registered IF image, and the generated mask image (positive regions are indicated in red), from left to right, respectively. The organ was shown above each triplet.

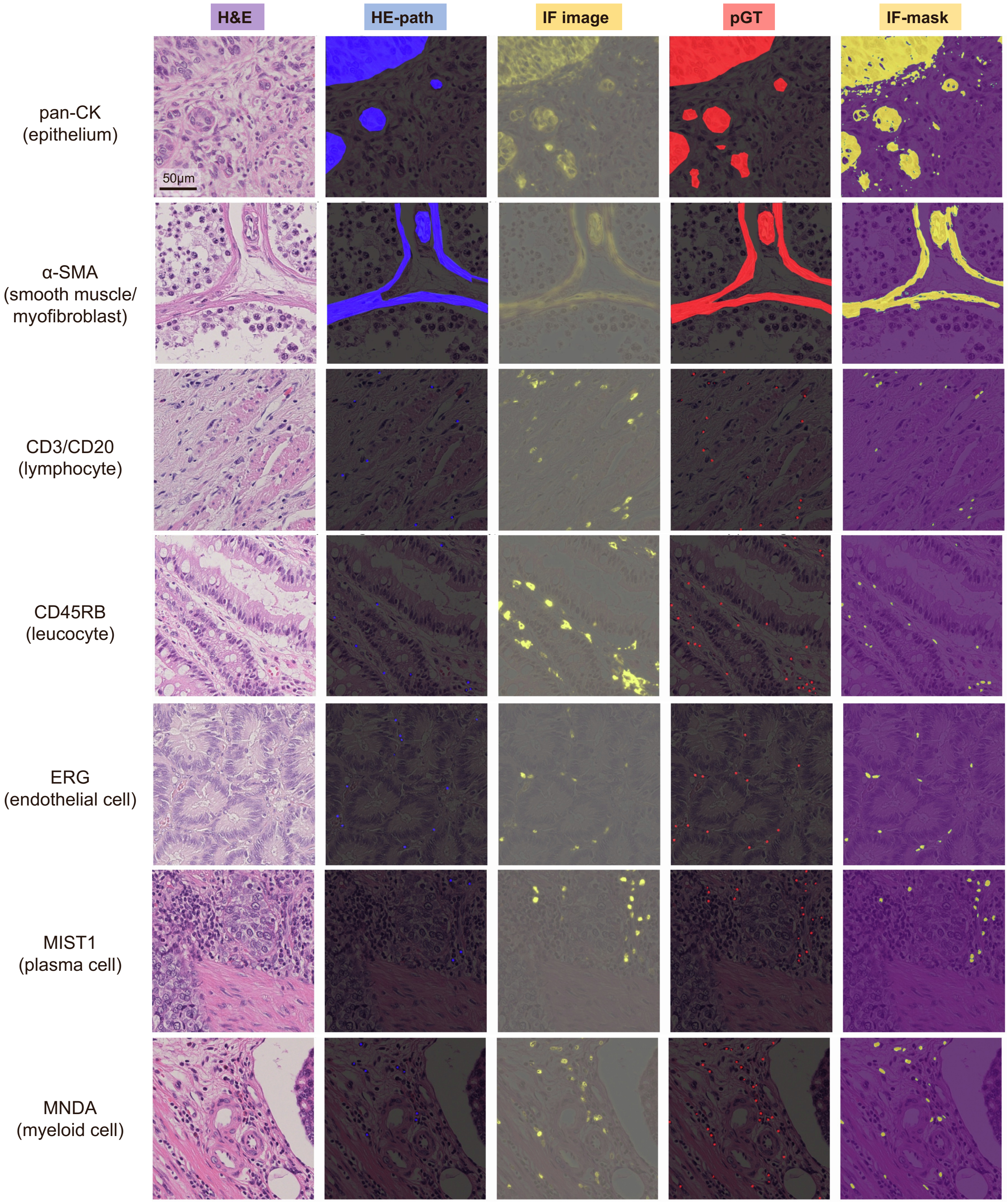

Figure S4. Annotation samples, related to Figure 4. H&E images (first column) and the corresponding annotations for pathologist annotations based on the HE-path images (second column), IF images (third column), HE-path and IF images (fourth column), and IF-mask (fifth column). From the second to fifth columns, the images are overlaid with the corresponding H&E images. The scales of all the image patches are the same.

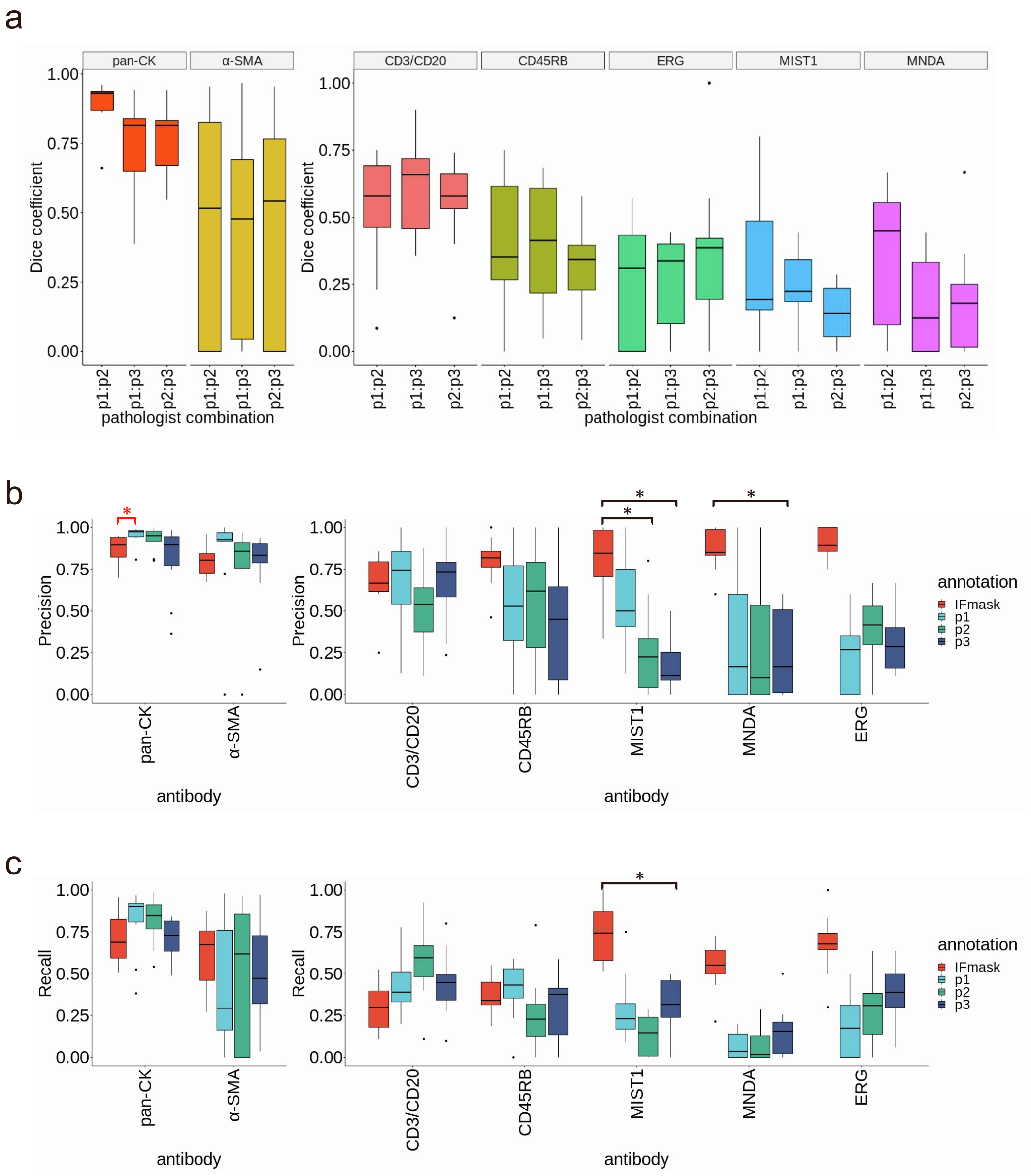

Figure S5. Evaluation of the annotation accuracy of SegPath, related to Figure 4. a, Inter-pathologist concordance of the annotations in terms of Dice coefficients (n = 10 patches of 217.5 × 217.5 μm for each tissue or cell type). Three pathologists annotated the tissues or cells based on the H&E images only. **b, c,** Comparison of the annotation accuracies between pathologists and the automatically generated masks in terms of precision shown in **b** and recall shown in **c**. (n = 10 patches of 217.5 × 217.5 μm for each tissue or cell type). pGT annotations were performed by pathologists who examined both the H&E and corresponding IF images. Regions or cells annotated by at least two of three pathologists were used. $*P < 0.05$.

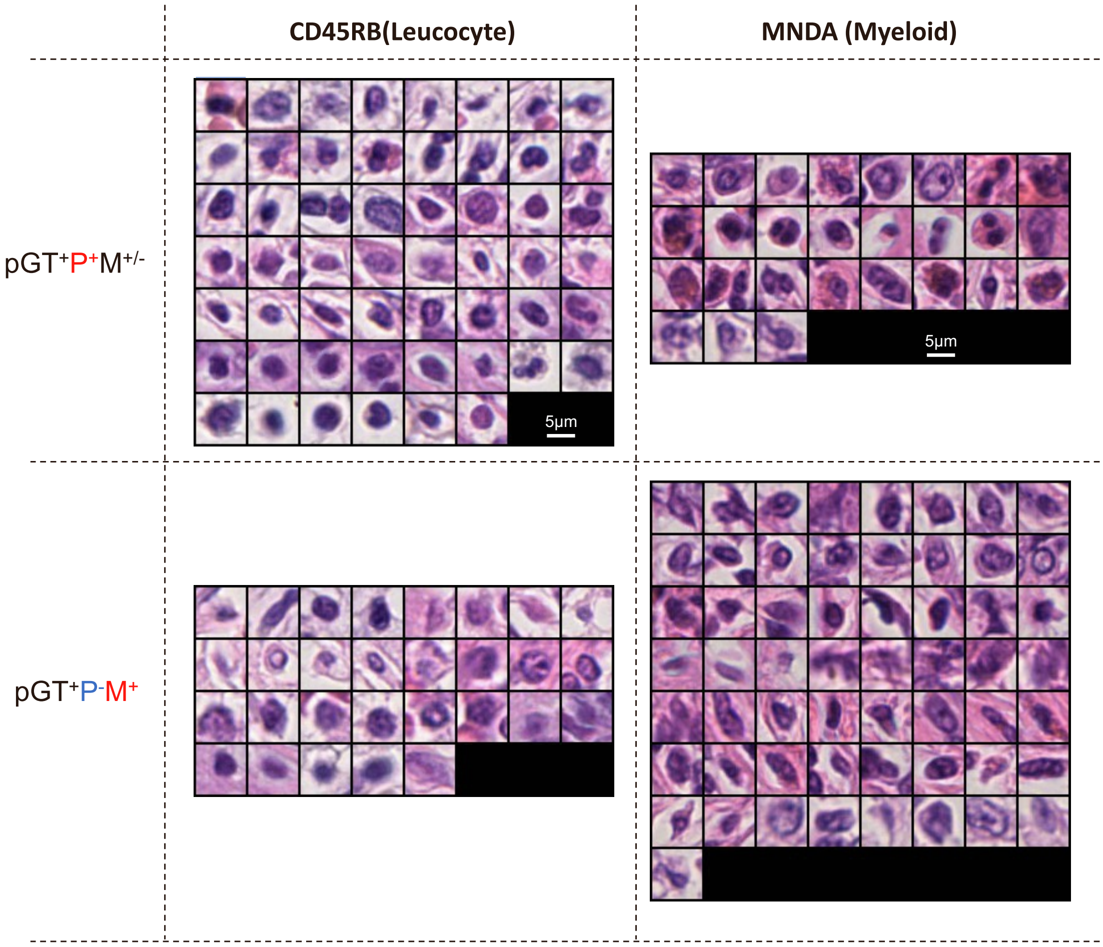

Figure S6. Annotated cell images for leucocytes and myeloid cells, related to Figure 4. pGT cell images annotated by multiple pathologists ($pGT^{+}P^{+}M^{+-}$) and not identified by multiple pathologists but successfully annotated using the masks ($pGT^{+}P^{-}M^{+}$). pGT, ground truth; P, HE-path; M, IF-mask.

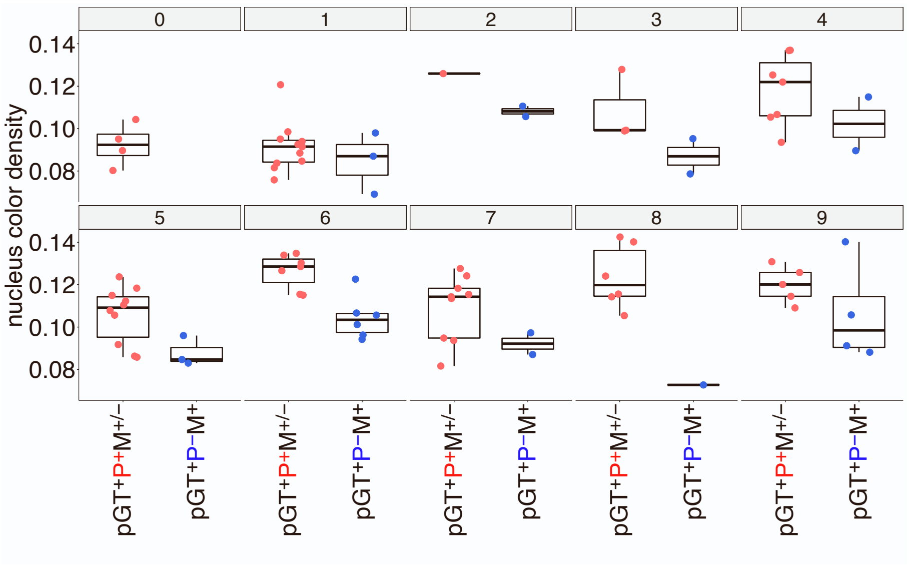

Figure S7. Nucleus haematoxylin intensity of lymphocytes for each patch, related to Figure 4. pGT, ground truth; P, HE-path; M, IF-mask.

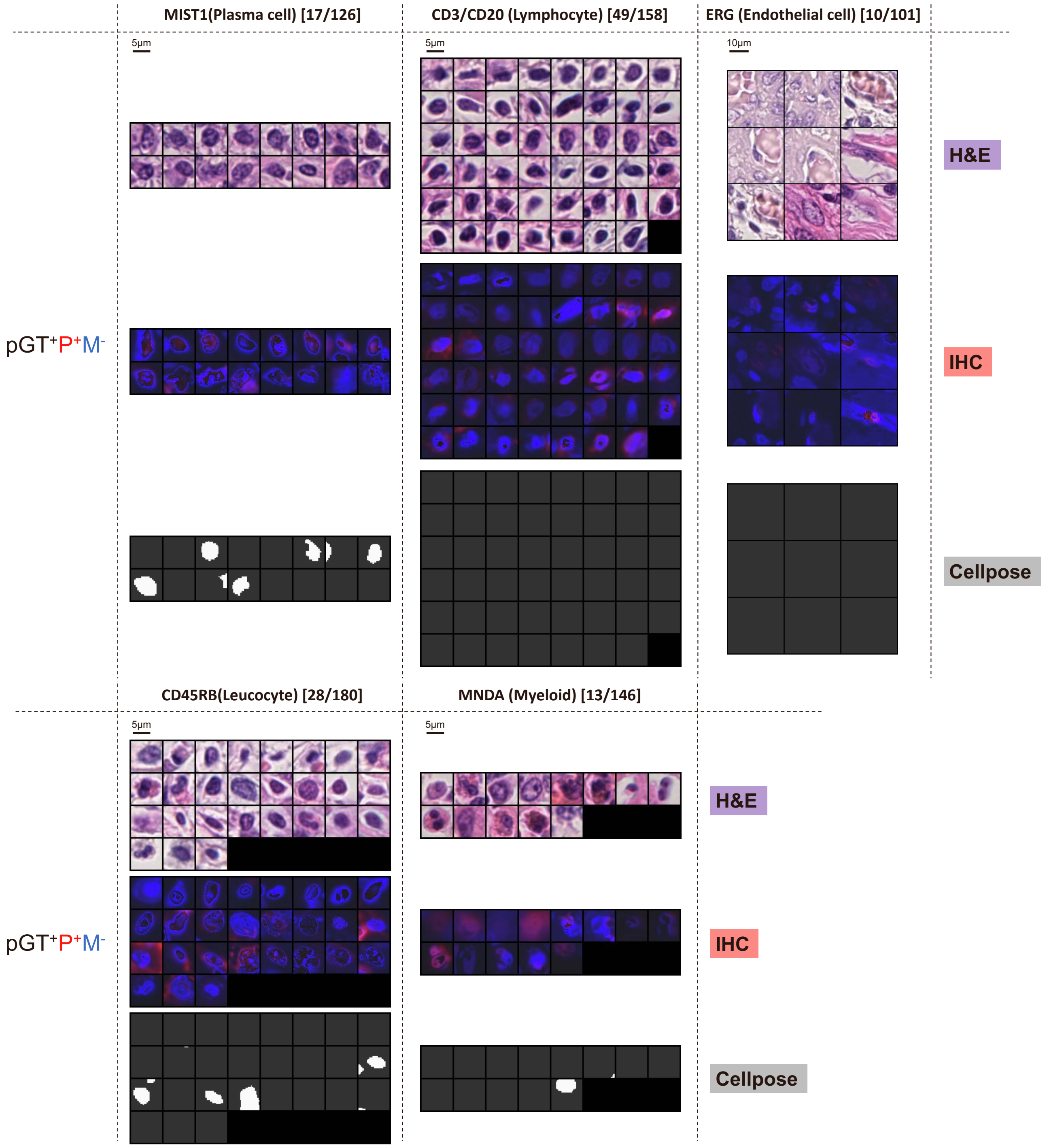

Figure S8. Error analysis of cells correctly identified by pathologists but missed by IF-masks, related to Figure 4. pGT cell images were annotated by multiple pathologists but were not identified by the masks ($pGT^{+}P^{-}M^{-}$). The corresponding IF images of the cell-specific antibodies (red), DAPI nuclear staining (blue), and nuclei detected by Cellpose (white) are shown. The numbers in brackets indicate the number of cells correctly identified by pathologists but missed when using the IF-masks, and the number of pGT cells. pGT, ground truth; P, HE-path; M, IF-mask.

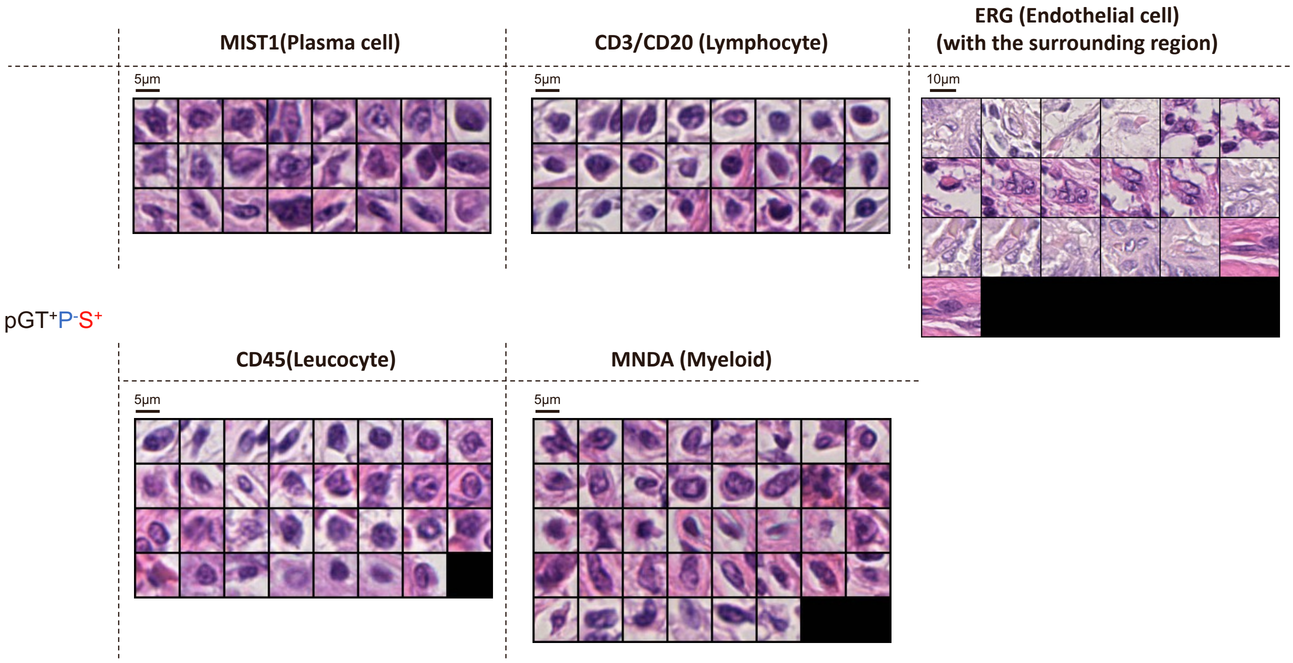

Figure S9. Annotated cell images, related to Figure 6. pGT cell images identified by multiple pathologists but not predicted when using the segmentation models (pGT$^+$P$^+$S$^-$). pGT, ground truth; P, HE-path; S, Prediction using the segmentation model. The scales of all the image patches for each cell type are the same.

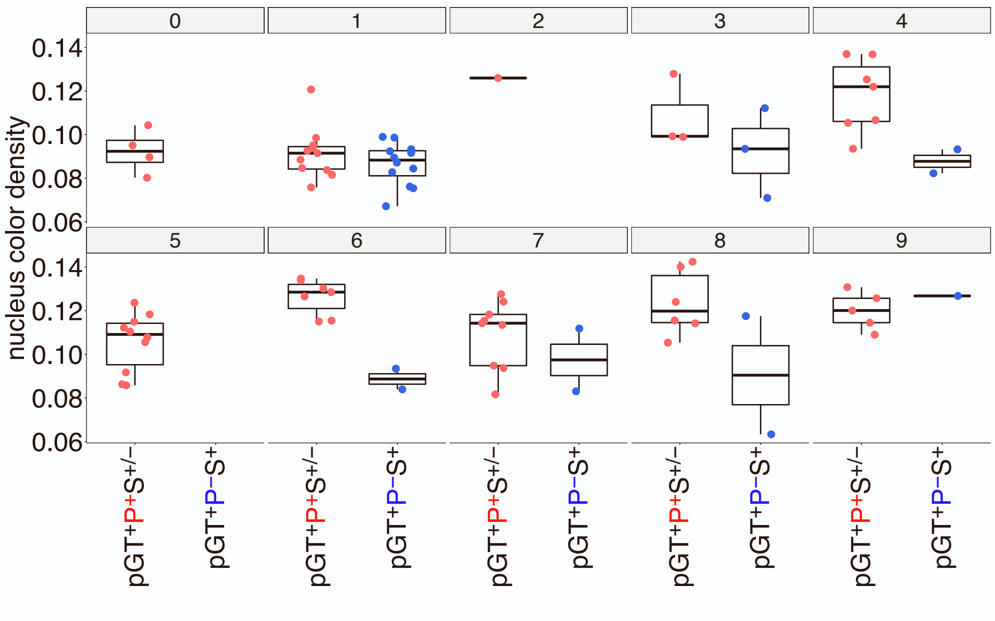

Figure S10. Nucleus haematoxylin intensity of lymphocytes, related to Figure 6. pGT, ground truth; P, HE-path; S, Prediction using the segmentation model.

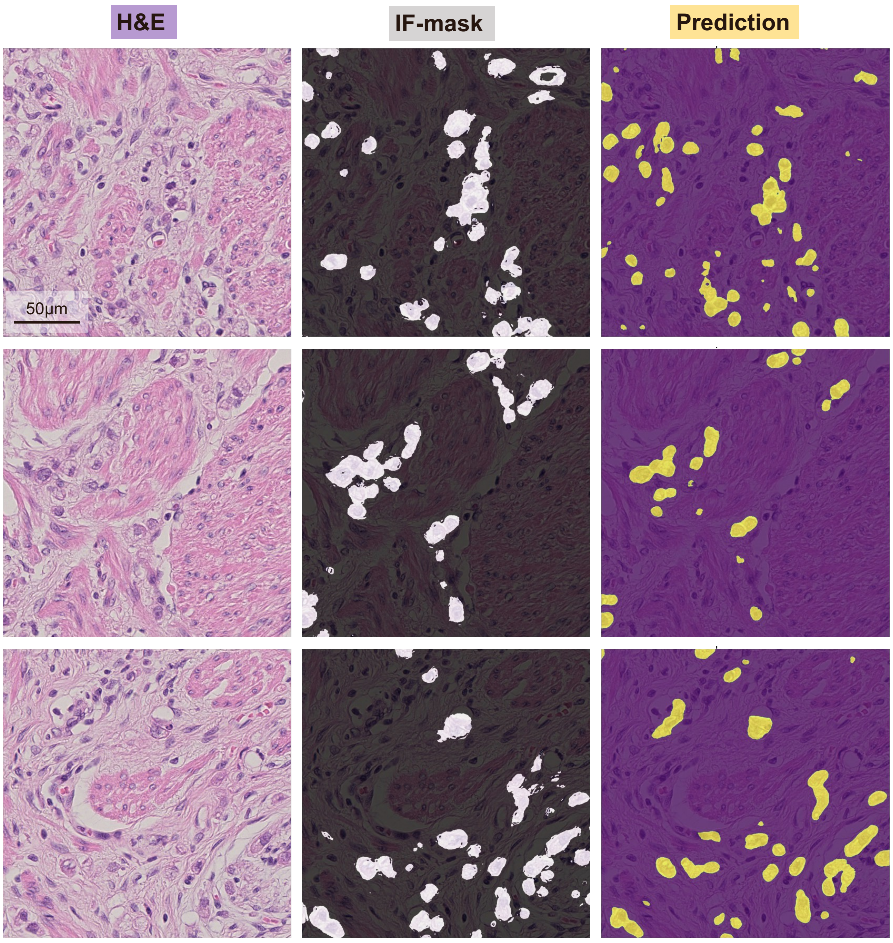

Figure S11. Isolated gastric cancer cells, related to Figure 6. HE-stained images (first columns), IF-masks (second columns), and predicted epithelium regions by the trained segmentation model (third columns) are shown for three different regions. The scales of all the image patches are the same.

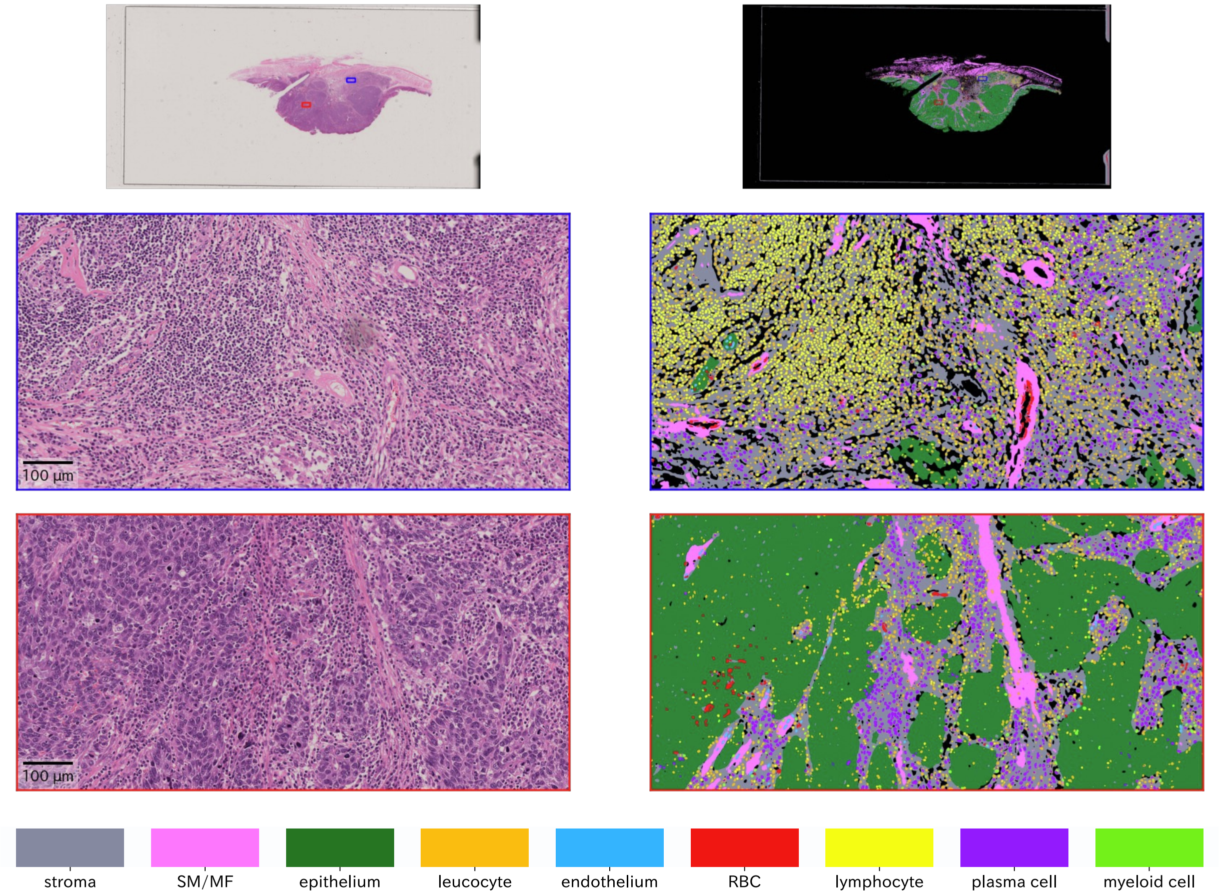

Figure S12. Visualisation of the multi-cell/tissue segmentation results by the trained segmentation models, related to Figure 6. WSI level segmentation of a gastric cancer specimen using the segmentation models. Regions in blue and red rectangles in the top WSI level image are enlarged in the middle and bottom images with a bounding box of the same colour. Different colours represent different cell types as indicated. Grey regions annotated as stroma were tissue regions not detected by any segmentation models. SM, smooth muscle; MF, myofibroblast.

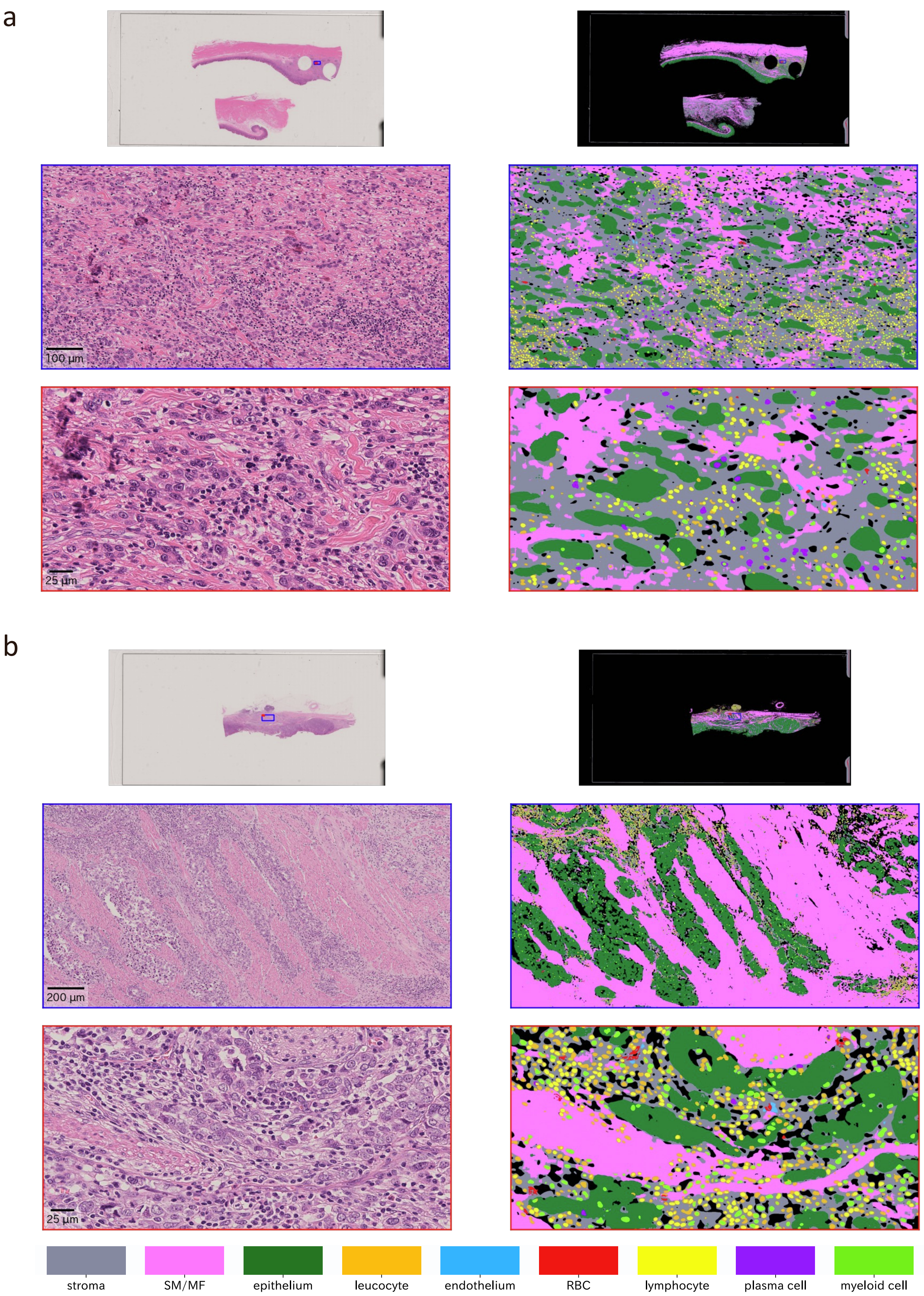

Figure S13. Visualisation of the multi-cell/tissue segmentation results by the trained segmentation models, related to Figure 6. a, b, Gastric cancer cases. SM, smooth muscle; MF, myofibroblast. The regions in the blue and red rectangles in the top WSI level image are enlarged in the middle and bottom images with a bounding box of the same colour.

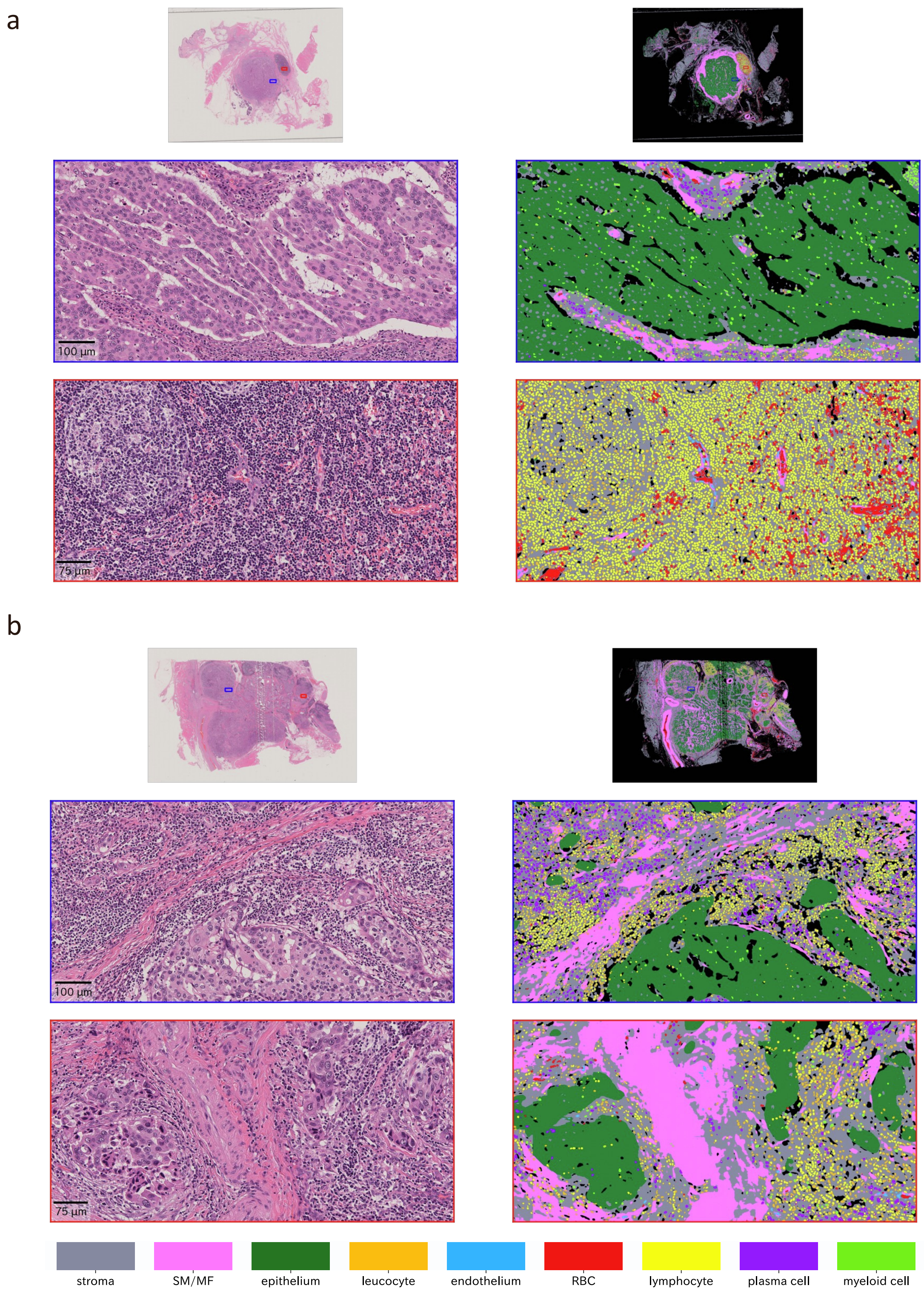

Figure S14. Visualisation of the multi-cell/tissue segmentation results by the trained segmentation models, related to Figure 6. Malignant salivary gland tumours. **a, b,** Salivary duct carcinoma cases. SM, smooth muscle; MF, myofibroblast. The regions in the blue and red rectangles in the top WSI level image are enlarged in the middle and bottom images with a bounding box of the same colour.

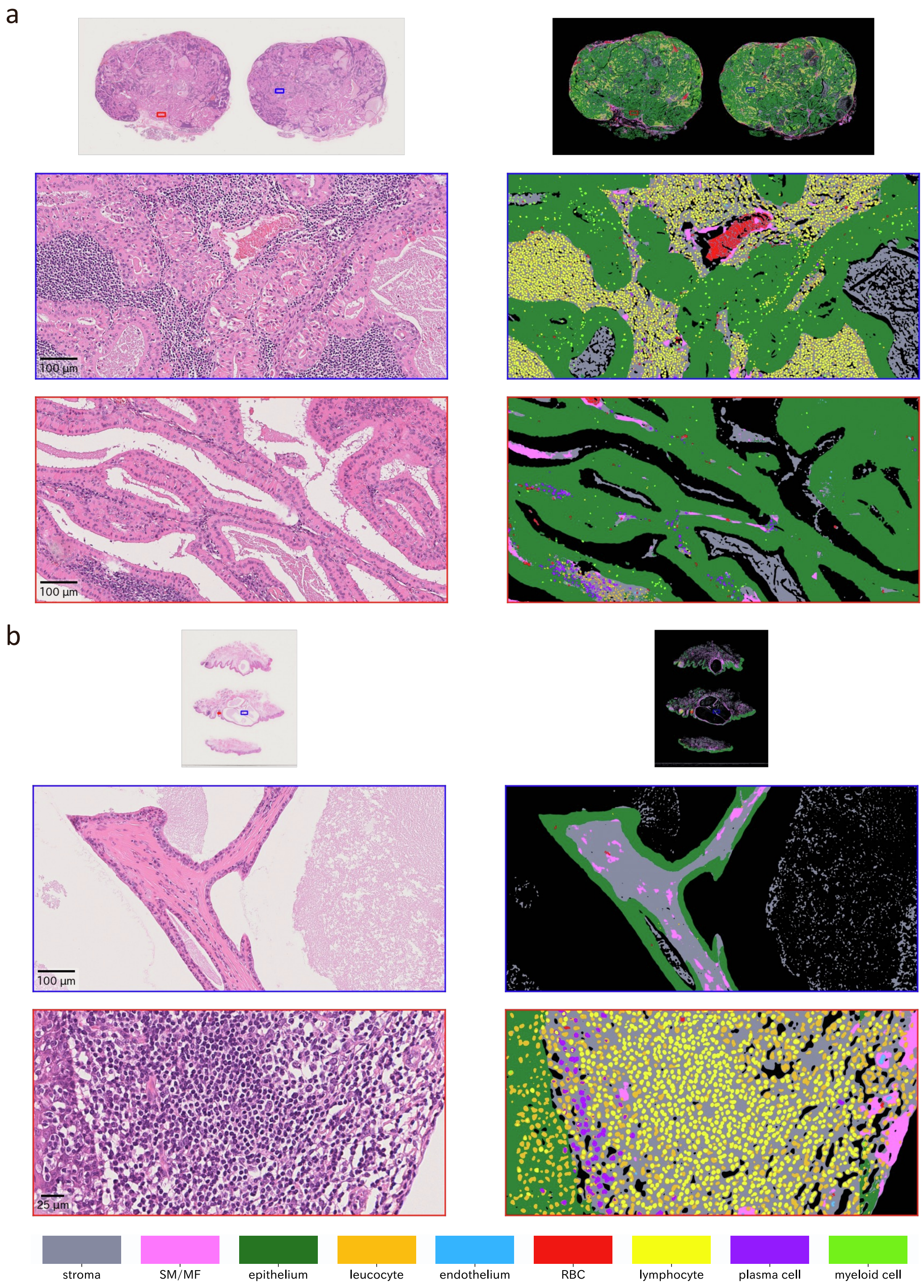

Figure S15. Visualisation of the multi-cell/tissue segmentation results by the trained segmentation models, related to Figure 6. Benign salivary gland tumours. **a,** Warthin's tumour. **b,** Cystadenoma. SM, smooth muscle; MF, myofibroblast. The regions in the blue and red rectangles in the top WSI level image are enlarged in the middle and bottom images with a bounding box of the same colour.

Table S1. Percentage of pixels changed by the threshold determined using the model with the second highest validation dice, related to Methods.

antigen	affected pixel (mean, %)	affected pixel (std, %)	affected pixel (median, %)
pan-CK	0.327	1.347	0
α-SMA	0.21	0.824	0
CD3/CD20	0.167	0.639	0.002
CD45RB	0.283	1.409	0.008
ERG	0.053	0.545	0
MIST1	0.072	0.764	0
MNDA	0.045	0.165	0

Table S4. Ranges of hyperparameters optimized with Optuna for the training of deep neural networks for segmentation, related to Methods.

Hyperparameter	Range	Distribution	Notes
loss function	combo (α*dice+(1-α)*focal loss); dice; binary cross entropy; focal tversky; focal loss; log cosh dice	categorical	
loss parameter	combo (α=[0.1-0.9]) ; focal tversky loss or focal loss (β=[3,8], γ=[0.5,3]); focal loss (β=10, γ=2)	uniform	
learning rate	[1E-4, 1E-2]	log uniform	
stochastic weight averaging	True;False	categorical	
model architeture	DeepLabV3Plus; UnetPlusPlus; Unet	categorical	
model_backbone	timm-efficientnet-b1; timm-efficientnet-b2; timm-efficientnet-b3; resnet18; resnet34; resnet50	categorical	
accumulated gradient	True; False	categorical	
encoder_weights	noisy student; imagnet	categorical	"noisy student" was aplied only when the efficient net was used

Table S6. The number of training patches or cells used in the analysis of sample size effect on the performance, related to Figure 5.

Antigen	Target	Target numbers of patches or cells#	unit
pan-CK	epithelium	100/500/1,000/5,000/10,000/15,000/21,912	patch
α-SMA	smooth muscle cell or myofibroblast	100/500/1,000/5,000/10,000/20,000/25,748	patch
CD235a	red blood cell	100/500/1,000/5,000/10,000/15,000/21,595	patch
CD45RB	leucocyte	100/500/1,000/5,000/10,000/50,000/100,000/250,000/500,000/727,503	cell
CD3/CD20	lymphocyte	100/500/1,000/5,000/10,000/50,000/100,000/250,000/329,792	cell
ERG	endothelial cell	100/500/1,000/5,000/10,000/25,000/50,000/56,229	cell
MIST1	plasma cell	100/500/1,000/5,000/10,000/25,000/50,000/72,259	cell
MNDA	myeloid cell	100/500/1,000/5,000/10,000/50,000/100,000/150,000/167,451	cell

The last number is the total number of patches or cells in the training dataset, thus only one test was performed.
