# Supplementary material for: Restaining-based annotation for cancer histology segmentation to overcome annotation-related limitations among pathologists
Source: Patterns (N Y). 2023 Feb 10;4(2):100688. doi: 10.1016/j.patter.2023.100688 (PMC9982301; doi:10.1016/j.patter.2023.100688)
Supplement: Document S2. Article plus supplemental information [file mmc5.pdf]

Patterns

Descriptor

Restaining-based annotation for cancer histology segmentation to overcome annotation-related limitations among pathologists

Graphical abstract

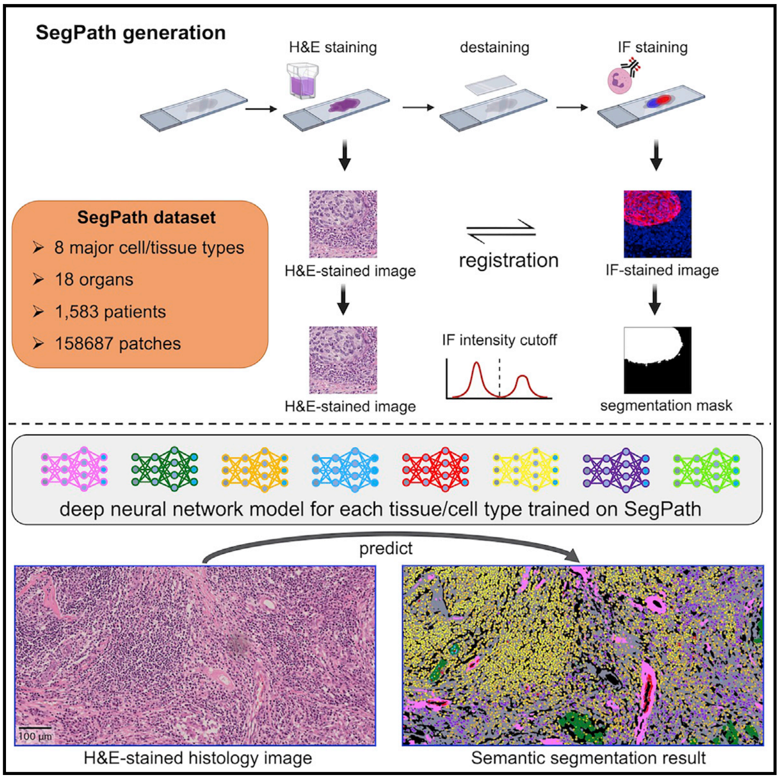

Highlights

- SegPath is the largest annotation dataset for cancer histology segmentation
- Immunofluorescence restaining enables high-throughput and accurate annotation
- SegPath is morphologically less biased than pathologists' annotation

Authors

Daisuke Komura, Takumi Onoyama, Koki Shinbo, ..., Tohru Ikeda, Tetsuo Ushiku, Shumpei Ishikawa

Correspondence

ishum-prm@m.u-tokyo.ac.jp

In brief

We created the largest-scale datasets for the segmentation of cancer histology images. Immunostaining with antibodies that recognize eight tissue/cell types yields datasets that are more accurate than those of conventional human annotations. These datasets enable the development of accurate deep-learning models for cancer histological images, which could assist in computer-aided diagnosis, interpretation of the diagnosis, and basic science of cancer.

Komura et al., 2023, Patterns *4*, 100688
February 10, 2023 © 2023 The Author(s).
https://doi.org/10.1016/j.patter.2023.100688

CellPress

Patterns

CellPress
OPEN ACCESS

Descriptor

Restaining-based annotation for cancer histology segmentation to overcome annotation-related limitations among pathologists

Daisuke Komura,[1] Takumi Onoyama,[1,2] Koki Shinbo,[1] Hiroto Odaka,[1] Minako Hayakawa,[1,3] Mieko Ochi,[1] Ranny Rahaningrum Herdiantoputri,[4] Haruya Endo,[1] Hiroto Katoh,[1] Tohru Ikeda,[4] Tetsuo Ushiku,[3] and Shumpei Ishikawa[1,5,6,*]

[1]Department of Preventive Medicine, Graduate School of Medicine, The University of Tokyo, 7-3-1 Hongo, Bunkyo-ku, Tokyo 113-0033, Japan

[2]Division of Gastroenterology and Nephrology, Department of Multidisciplinary Internal Medicine, School of Medicine, Faculty of Medicine, Tottori University, 36-1 Nishicho, Yonago, Tottori 683-8504, Japan

[3]Department of Pathology, Graduate School of Medicine, The University of Tokyo, 7-3-1 Hongo, Bunkyo-ku, Tokyo 113-0033, Japan

[4]Department of Oral Pathology, Graduate School of Medical and Dental Sciences, Tokyo Medical and Dental University, 1-5-45 Yushima, Bunkyo-ku, Tokyo 113-8549, Japan

[5]Division of Pathology, National Cancer Center Exploratory Oncology Research & Clinical Trial Center, 6-5-1 Kashiwanoha, Kashiwa, Chiba 277-8577, Japan

[6]Lead contact

*Correspondence: ishum-prm@m.u-tokyo.ac.jp

https://doi.org/10.1016/j.patter.2023.100688

THE BIGGER PICTURE Tumor tissue is composed of various cell types. Information on the location of various cells in tumor tissue is essential to identifying tumor features; however, the accurate and quick estimation of this information is challenging. Deep-learning-based segmentation can overcome this challenge but is hindered by the insufficient amount of training data. We therefore created training datasets for the segmentation of various tissues or cells at an unprecedented scale through immunostaining with antibodies that identify various tissue/cell types. SegPath annotation outperforms manual annotation in terms of accuracy and morphological bias, leading to more optimized segmentation model development. Application of the segmentation model trained on SegPath to a large number of cancer histopathology specimens that have been accumulated in hospitals could significantly impact cancer diagnosis and acquisition of additional insight into cancer research.

1 2 3 4 5 **Proof-of-Concept:** Data science output has been formulated, implemented, and tested for one domain/problem

SUMMARY

Numerous cancer histopathology specimens have been collected and digitized over the past few decades. A comprehensive evaluation of the distribution of various cells in tumor tissue sections can provide valuable information for understanding cancer. Deep learning is suitable for achieving these goals; however, the collection of extensive, unbiased training data is hindered, thus limiting the production of accurate segmentation models. This study presents SegPath—the largest annotation dataset (>10 times larger than publicly available annotations)—for the segmentation of hematoxylin and eosin (H&E)-stained sections for eight major cell types in cancer tissue. The SegPath generating pipeline used H&E-stained sections that were destained and subsequently immunofluorescence-stained with carefully selected antibodies. We found that SegPath is comparable with, or outperforms, pathologist annotations. Moreover, annotations by pathologists are biased toward typical morphologies. However, the model trained on SegPath can overcome this limitation. Our results provide foundational datasets for machine-learning research in histopathology.

This is an open access article under the CC BY license (http://creativecommons.org/licenses/by/4.0/).

INTRODUCTION

Tumor tissues comprise various cell types, each with a unique function and morphology. In cancer histopathology, information on cell components and their distribution in the tumor tissues of patients aids with diagnoses, classification of tumor subtypes, prediction of prognosis and therapeutic effects, and understanding the underlying mechanisms of carcinogenesis.[1,2] Although pathologists estimate such information in clinical practice, the quantitative and comprehensive measurement of cell components and distribution data is almost impossible, particularly for large tissue specimens. Therefore, an automatic segmentation system using routinely used hematoxylin and eosin (H&E)-stained tumor sections can be highly valuable in medical practice and cancer research.

Deep neural networks are emerging machine-learning technologies capable of performing such tasks with remarkable accuracy.[3–6] However, the remarkable performance of deep neural networks is attributed to their abundant annotations, which are often difficult to obtain in medical imaging. There are large-scale publicly available datasets for the semantic segmentation of H&E images based on numerous efforts to annotate tissues or cells, most of which rely on human annotators.[6,7] For example, GlaS[8] is a dataset of colorectal gland segmentation consisting of 165 images derived from 16 histological sections annotated by a single pathologist. BCSS[9] contains more than 20,000 tissue annotations for segmentation and NuCLS[7] contains 220,000 cell annotations for detection or segmentation, both from breast cancer images. These two datasets were annotated by a non-pathologist and then refined by multiple pathologists to increase the scale of the dataset. In addition, Camelyon[10] annotated 499 H&E slides with pathologist-annotated boundaries of metastatic breast cancer cells, some of which were confirmed by cytokeratin immunohistochemistry (IHC) on serial sections. CoNIC[5] is the largest dataset to date for the segmentation of six types of nuclei from colon cancer, incorporating artificial intelligence to perform the annotation; however, the difficult cases are annotated by a pathologist. MoNuSAC2020[11] comprises 31,000 nuclear boundary annotations for epithelia, lymphocytes, macrophages, and neutrophils from four organs (lung, prostate, kidney, and breast).

These datasets facilitate the development of deep-learning models for cancer tissue/cell segmentation or detection. However, manual annotation of tumor tissues by non-pathologists is not feasible and is considerably time and labor intensive, thereby limiting the generation of large-scale annotated datasets that cover more tissue/cell and tumor types. Another key issue that has often been overlooked in previous research is the fact that human annotations may not cover the full diversity of cell morphologies. Cells do not always have the typical morphologies described in textbooks. The surrounding environments (e.g., narrow lumen) can deform cells, which may lead to the presentation of atypical morphologies depending on the location and angle of the cell cross-section. The morphologies of cells can also be altered by molecular interactions with the surrounding microenvironment. For example, the identification of tumor vascular endothelial cells may be complicated by their enlarged nuclei and morphologies, which are similar to those of epithelial cells.[12] Additionally, the accurate identification of certain cell types, such as myeloid cells, by pathologists can be complicated, as evidenced by the high rates of macrophage count discordance among pathologists.[13] Such factors inhibit the accurate annotation of cells with atypical morphologies by pathologists and potentially limit the performance of segmentation models trained using the datasets.

Recently, new methods using IHC technologies or special stains for the annotation of histological images have emerged to overcome the aforementioned limitations.[14–17] Ing et al.[14] used an anti-CD31 antibody to stain vascular endothelial cells in 204 destained H&E slides of renal cancer and developed a segmentation model for vascular endothelial cells. In addition, Liu et al.[15] used Ki-67 IHC staining to stain proliferating cells in 12 destained H&E slides to develop a model for detecting proliferating cells in a neuroendocrine tumor, and Bulten et al.[17] created a dataset for epithelium segmentation of 102 H&E-stained prostate specimens using IHC-restained images as a guide for generating masks. This approach is powerful because the restaining procedure can produce perfectly matched slides instead of consecutive slides, enabling the formation of accurate segmentation masks without the need for pathologist intervention. However, such datasets are not publicly available or are limited with regard to cell types and tissues.

Our study adopted the aforementioned approach by creating a dataset for the semantic segmentation of various tissues or cells at an unprecedented scale. We developed an annotation workflow with minimal pathologist intervention based on H&E-stained sections that were destained and immunofluorescence (IF) stained. Because IF relies on the proteins expressed in target cells, it can capture the target cells with diverse morphologies in a more optimized manner than human annotations. Using carefully selected antibodies with high specificities for each of the eight major constituent cells in tumor tissues, we generated SegPath, a high-quality dataset of diverse cell types. SegPath is the largest annotated tissue and cell segmentation dataset of H&E images of various organs. SegPath has been made accessible to the public (https://dakomura.github.io/SegPath) to contribute to the development of new segmentation models.

RESULTS

Dataset generation workflow

The workflow for creating SegPath is shown in Figure 1A. First, tissue microarray (TMA) sections prepared from well-preserved formalin-fixed paraffin-embedded (FFPE) tissues were stained with H&E using a standard procedure. They were then digitized using a slide scanner to create whole-slide images (WSIs) at 40× resolution. After destaining the H&E-stained sections with alcohol and autoclave processing, IF and 4′,6-diamidino-2-phenylindole dihydrochloride (DAPI) nuclear staining was performed using antibodies that specifically recognized each cell type. The sections were then digitized again. The procedures were performed within a few days to prevent the degradation of IF staining.[18] After IF staining, the pathologists confirmed that the staining quality was sufficiently high. Multi-resolution rigid registration between the H&E and IF images was performed to ensure that the alignment of the hematoxylin component in the H&E images and DAPI in the IF images, both recognizing nuclei. Registration was first performed at the WSI level and then at the patch level. After rigid registration, a few cells shifted locally and slightly

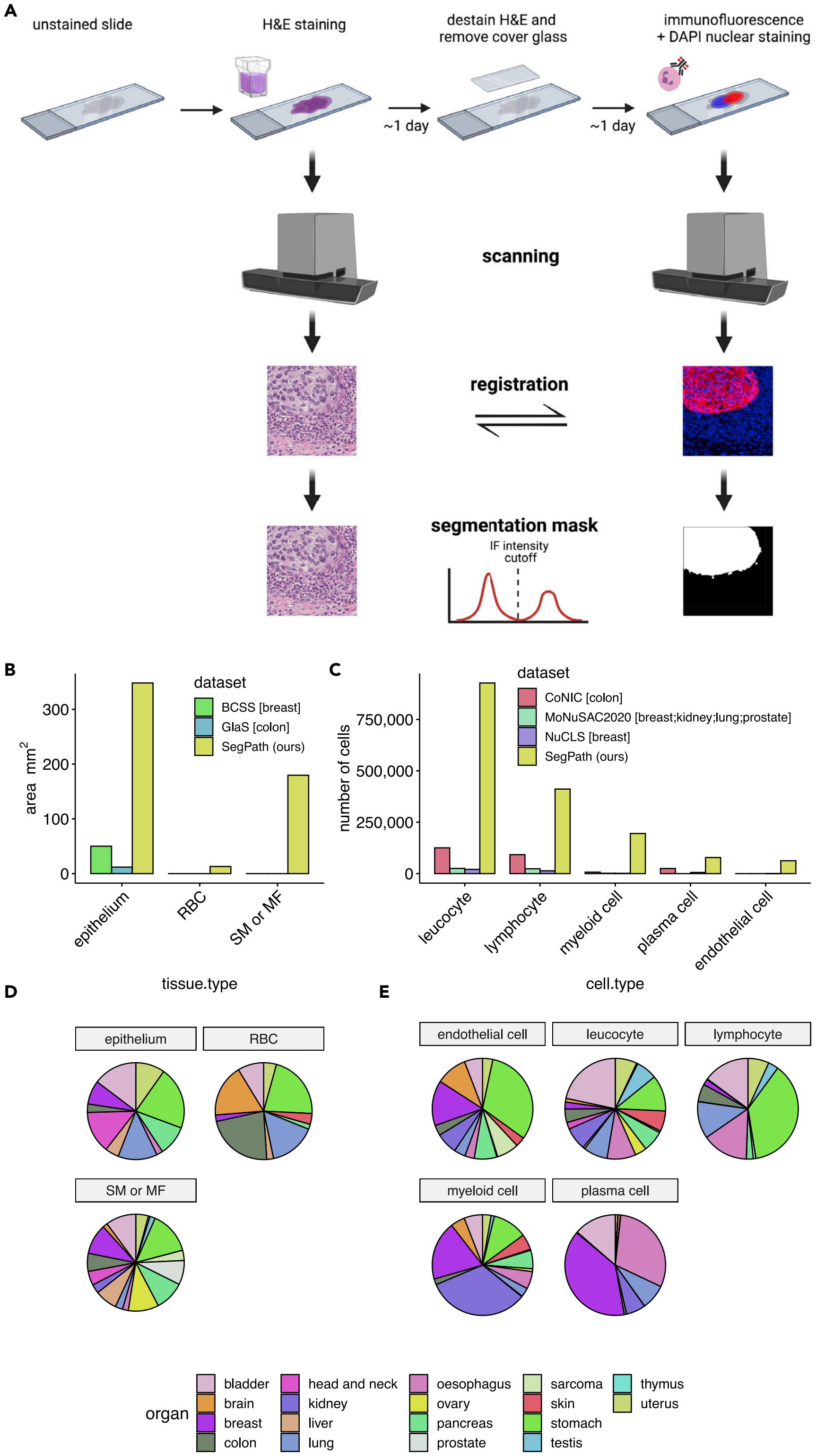

Figure 1. Generation of annotation masks for tissue/cell-type segmentation using IF restaining

(A) Workflow overview. After scanning the H&E-stained sections, the sections were destained and restained with DAPI nuclear staining and IF staining with target-specific antibodies. The slides were then scanned, and the positions of the two slides were aligned with registration algorithms. Small patches were created. Cut-off values of IF signal intensity were determined for each patch to generate segmentation masks in an iterative manner based on the segmentation results of the deep neural network training on the generated masks. For endothelial cells, leukocytes, lymphocytes, myeloid cells, and plasma cells, a nucleus detection algorithm was applied to the DAPI channel. Positive signals of the target cell in IF were transferred to the corresponding nuclei. See also Figures S1 and S2.

(B and C) (B) Annotated areas for each tissue and (C) the number of annotated cells for each cell type in SegPath. Those in publicly available datasets, including BCSS,[9] GlaS,[8] CoNIC,[5] MoNuSAC2020,[11] and NuCLS,[7] are also shown. Organs in brackets represent the target organs of the dataset. "SM" or "MF" include all stroma in the GlaS and BCSS datasets.

(D) Distribution of target organs in SegPath.

(E) Distribution of cell types in SegPath. SM, smooth muscle cell; MF, myofibroblast.

See also Table S2.

in inconsistent directions during destaining and restaining. Therefore, we performed an additional non-rigid registration for each patch to accurately align the nuclei (Figure S1A). We also observed tissue folding in certain slides during destaining and restaining, but pathologists carefully annotated them in WSI and removed the regions in the patch selection process.

Subsequently, we created a binary segmentation mask based on the IF images (hereafter referred to as IF-mask) (Figure S2). The area where the fluorescence intensity exceeded the cut-off value initially determined manually was labeled positive. The false positives derived from red blood cell (RBC) autofluorescence estimated using the deep neural network, which was trained on the dataset using an anti-CD235a antibody recognizing RBCs, were labeled negative in the non-RBC datasets. For the hematopoietic and endothelial cells, the positive regions of the target cells were transferred to the cell nuclei to reduce false positives and make the segmentation task more traceable. Therefore, we used Cellpose,[19] a pre-trained deep neural network model for nuclear segmentation, with the DAPI images to identify the nuclei (Figure S1B). We then labeled whole nuclei as positive if the positive region overlapped with the nuclei over a certain level. Subsequently, the patches were divided into training, validation, and test datasets. Finally, we iteratively

Table 1. Antibodies used in this study

Antigen	Clone	Host	Target	Localization	Company	Product no.	Evidence[a]
Pan-cytokeratin (pan-CK)	AE1/AE3	mouse	epithelium	cytoplasmic	DAKO	IS05330-2J	used in clinical practice
CD3	polyclonal	rabbit	T lymphocyte	cell membrane, cytoplasmic	DAKO	IS50330-2J	used in clinical practice
CD20	L26	mouse	B lymphocyte	cell membrane, cytoplasmic	DAKO	IS60430-2J	used in clinical practice
CD45RB	2B11+PD7/26	mouse	Leukocyte	cell membrane, cytoplasmic	DAKO	IR75161-2J	used in clinical practice
αSMA	1A4	mouse	smooth muscle/ myofibroblast	cytoplasmic	DAKO	M085129-2	used in clinical practice
ERG	9FY	mouse	blood vessel, lymphatic vessel	nuclei	Biocare Medical	PM421AA	PMID: 23334893
MIST1	D7N4B	rabbit	plasmacyte	nuclei	Cell Signaling Technology	#14896	PMID: 22495370
MNDA	polyclonal	rabbit	myeloid cell	nuclei	Sigma-Aldrich	HPA034532-100UL	https://www.proteinatlas.org/ENSG00000163563-MNDA/antibody
Glycophorin A (CD235a)	JC159	mouse	erythrocyte	cell membrane	Thermo Fisher Scientific	MA5-12484	PMID: 24399013

[a]Supporting evidence of sensitivity/specificity.

improved the fluorescence intensity threshold using deep neural network models, as the intensity gradient was observed in the same WSIs, possibly owing to uneven antibody concentrations during staining; therefore, the fixed threshold was not optimal. The deep neural network model for the target tissue/cell was trained on the training dataset with annotations using the fluorescence intensity threshold in the iteration. Otsu's threshold[20] for successfully segmented patches with positive regions, where there was a positive correlation between the IF density and prediction probability, was used as the cut-off value in the subsequent iteration. For the other patches, the threshold was the weighted mean of the cut-off value of the neighboring successfully segmented patches, where the weight was determined based on the pixel distance between patches. This process was repeated twice until the generated IF-masks had been converged (Figure S1C). We confirmed that the different segmentation models with similar performance in terms of validation loss flipped only 0.045%–0.327% of pixels in the segmentation mask on average (Table S1).

Nine different antibodies, including five antibodies used in clinical practice, were used to cover the main cell components of the tumor tissue (Table 1). A mixture of anti-CD3 and anti-CD20 antibodies was used for lymphocytes. Because our workflow can generate segmentation masks in a high-throughput manner without the need for manual annotation, the size of the dataset was over one order of magnitude larger than the currently available segmentation mask datasets for tumor tissues[5,7–9,11] (Figures 1B and 1C). In addition, we created datasets for as many as 18 different organs from 1,583 patients (Figures 1D and 1E) to cover a wider spectrum of cancer types (Table S2) than in the currently available datasets, which cover up to four organs. Finally, our SegPath dataset consists of 158,687 patches of 984 × 984 pixels at 40× resolution. Dataset statistics, including train/validation/test splits, are shown in Tables 2 and S3.

Antibody and organ selection

The choice of antibodies is one of the most important factors in the successful generation of IF-masks. We carefully selected the proteins that are specifically expressed in the target cell types based on the gene expression profiles (Figure 2A). Moreover, we selected cytoplasmic proteins for tissue-type segmentation (epithelium and smooth muscle cell/myofibroblasts). For hematopoietic cells or the endothelia, we prioritized proteins localized in the nuclei of target cell types because the position of the cells can be easier to identify with the antibody to such proteins. For lymphocytes and leukocytes, we selected antibodies used in clinical practice that stained the cell membrane, mainly because the appropriate antibodies that localized to the nuclei could not be found despite various trials using candidate antibodies (data not shown).

We observed several failures during the antibody selection process. A few of the antibodies that had been initially selected exhibited low staining intensities or specificities, depending on the clone. In other cases, such as that with myeloperoxidase (MPO), the antigen diffused into the surrounding area, thereby complicating the accurate identification of the cell locations (Figure 2B). Although MIST1 is a plasma-cell-specific antigen, it was slightly stained with the selected anti-MIST1 antibody in certain glandular epithelial cells (Figure 2C). Therefore, organs such as the stomach, pancreas, and salivary glands were excluded from the MIST1 dataset. For the endothelium, although a few prostate cancer cases with ERG rearrangement could be positive in ERG staining, we confirmed that the prostate cancer cases in our cohort were negative in ERG staining. After optimizing the antibodies, the trained pathologists carefully

CellPress
OPEN ACCESS

Table 2. Dataset summary

Antigen	Target	Data partition	Tissue	Slide	Patient	Patches
Pan-CK	epithelium	Train	16	20	341	21,912
		Validation	16	19	34	2,259
		test	16	20	32	2,338
αSMA	smooth muscle/myofibroblast	train	27	27	419	25,748
		validation	25	25	40	2,489
		test	27	27	47	2,941
CD3/CD20	lymphocyte	train	22	28	244	10,453
		validation	15	18	24	1,082
		test	11	14	19	738
CD45RB	leukocyte	train	30	30	428	20,518
		validation	25	25	36	1,988
		test	25	25	41	2,299
ERG	blood /lymphatic vessel	train	22	24	256	9,497
		validation	10	11	14	613
		test	8	9	12	537
MIST1	plasma cell	train	20	37	310	11,320
		validation	14	19	23	947
		test	10	15	18	964
MNDA	myeloid cell	train	28	29	339	12,315
		validation	15	15	19	894
		test	16	17	20	926
CD235a	red blood cell	train	13	17	302	21,595
		validation	13	17	31	2,224
		test	13	17	33	2,090

See also Table S3.

confirmed that all the antibodies had high enough sensitivity and specificity for the target tissue or cells in the target organs by comparing H&E-stained images and the corresponding IF-stained images. In the process, prostate cancer, renal cancer, and hepatocellular carcinoma cases were removed from the pan-CK dataset owing to weak IF staining of tumor cells.

Dataset evaluation

Figure 3 shows examples of the H&E-stained images, matched IF images, and generated IF-masks in SegPath for the selected antibodies in various organs. For example, the anti-pan-CK antibody stained cytokeratin, which was localized in the cytoplasm of the epithelial cells. Although the nuclei of the epithelial cells were unstained in certain cells, the borders of the epithelial tissue regions were clear, indicating the use of the mask for epithelial segmentation. The anti-α-smooth muscle actin (αSMA) antibody stained perivascular smooth muscle cells densely and smooth muscle or myofibroblasts in some stroma less densely (Figure S3), which possibly reflected the density and expression of αSMA (e.g., cancer-associated fibroblasts [CAFs], which differentiate into cells with the myofibroblast phenotype, are morphologically similar to smooth muscle cells but have variable αSMA expression depending on the degree of differentiation). Anti-CD45RB and anti-CD3/CD20 antibodies recognized the proteins on the cell membranes of leukocytes and lymphocytes, respectively; however, additional pre-processing using a nucleus detection algorithm caused the generated masks to cover the nuclei only, thereby clarifying the cell positions. The masks were almost identical to the IF images for the antibodies against ERG, myeloid cell nuclear differentiation antigen (MNDA), and MIST1.

To quantitatively evaluate the quality of the IF-masks in SegPath, we compared them with two types of manual annotations: the annotation created by three trained pathologists evaluating the H&E images alone (hereafter referred to as HE-path), and both the H&E and corresponding IF images (pathologist-guided ground truth, hereafter referred to as pGT) (Figures 4 and S4). The evaluation dataset consisted of 20 image patches of 217.5 × 217.5 μm for each antibody. The pathologists generated HE-paths based on morphology and pGTs based on morphology and IF intensity and distribution. The regions or cells annotated by at least two pathologists were regarded as positive. Therefore, the HE-paths may be considered as baselines in conventional manual annotations, and the pGTs can be considered to be closest to the ground truth, as pathologists are thought to be less affected by spurious IF signals.

First, we examined the concordance of the HE-paths among the three pathologists (Figure S5A). The concordance varied immensely depending on the tissue and cell type. It was nearly identical in terms of epithelial tissues but showed a little overlap in the endothelia, plasma cells, and myeloid cells. These

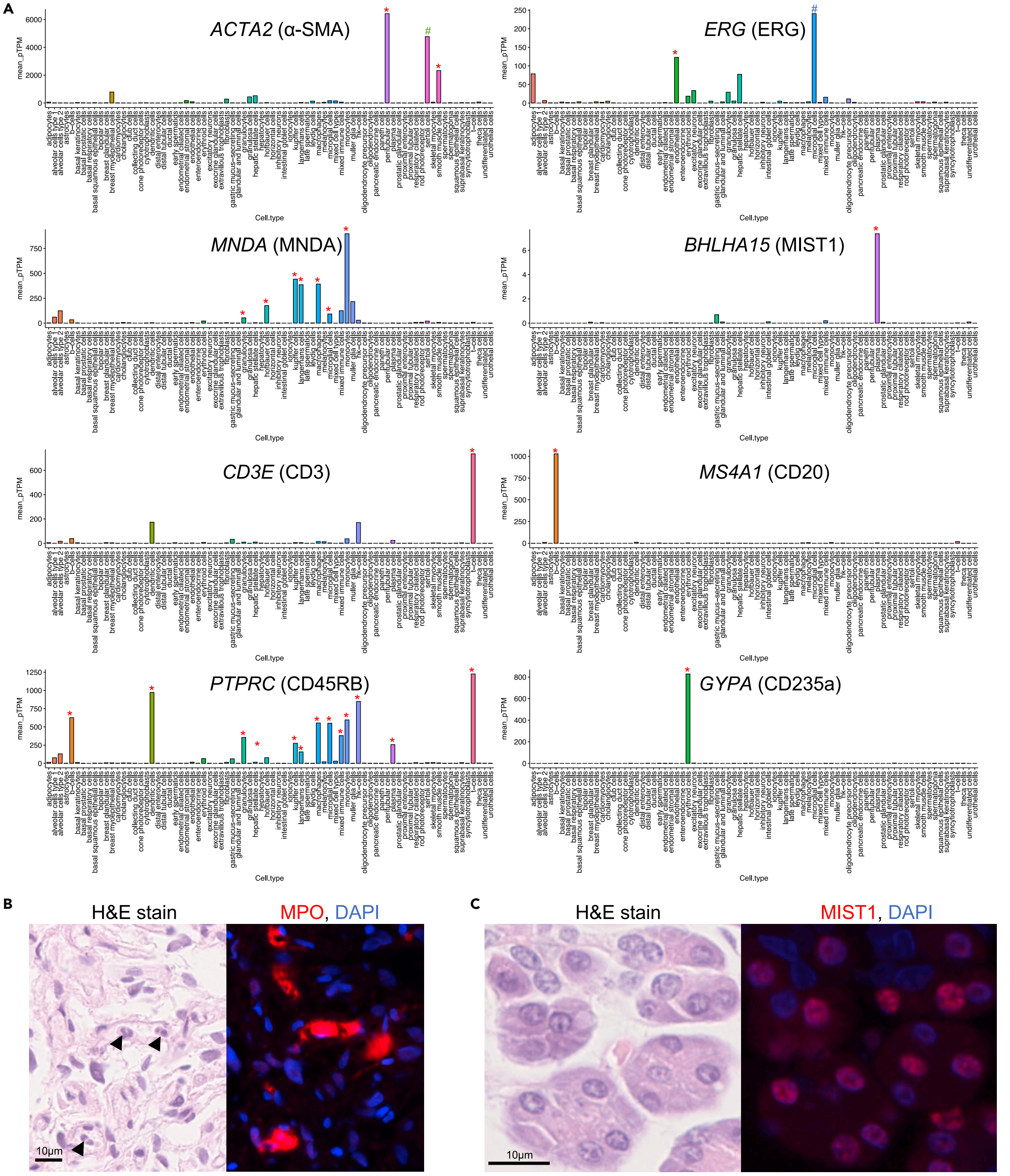

Figure 2. Selection of the antibodies and target tissues in SegPath

(A) Gene expression specificities of selected antibodies. Gene expression data were retrieved from single-cell transcriptome profiles in the Human Protein Atlas.[21,22] Target cell type is indicated by a red asterisk on the bar. *ACTA2* expression in Sertoli cells, indicated by a green octothorpe, was high in this dataset, but a pathologist could not confirm the positive staining of anti-α-smooth muscle actin (SMA) antibody; therefore, testicular tissues were included in the dataset. *ERG* expression in microglial cells, indicated by a blue octothorpe, was higher in this dataset. This is highly likely to be an erroneous annotation of the single-cell transcriptome profile, as confirmed by a pathologist; therefore, brain tissues were included in the dataset.

(legend continued on next page)

CellPress
OPEN ACCESS

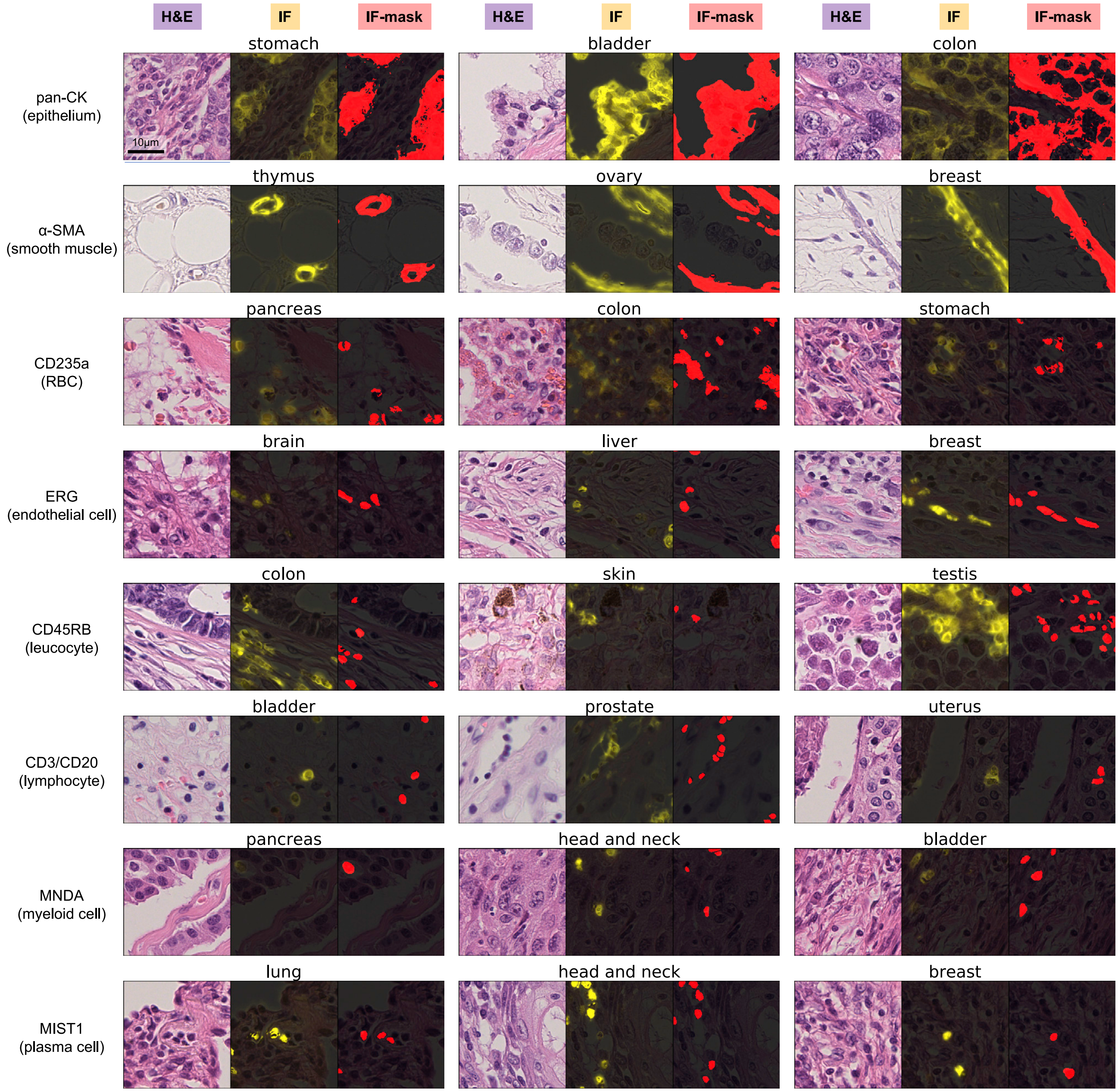

Figure 3. Generated masks in cancers of various organs

Each triplet shows an H&E-stained image, the corresponding registered IF image, and generated mask image (positive regions are indicated by red) from left to right, respectively. The organs are shown above each triplet. All image patches are 72.5 × 72.5 μm. See also Figure S3.

observations highlighted the complexity of accurate cell identification by pathologists.

Next, we evaluated the correctness of the HE-paths and IF-masks of each tissue or cell type in terms of the Dice coefficient (F1 score), precision, and recall (Figures 4A, S5B, and S5C) indices compared with those of the pGTs. We found that the performance of the HE-paths for the five cell types was low, indicating that it would be difficult for pathologists to identify such cells accurately. Conversely, the IF-masks were significantly more accurate than the HE-paths, especially in plasma

(B) H&E-stained image and IF staining of anti-MPO antibody, which targets neutrophils. Antigens spread around the target cells, as indicated by arrowheads, prevent accurate mask generation.

(C) IF staining of anti-MIST1 antibody, which targets plasma cells. It unexpectedly stained the nuclei of some glandular epithelia, including the salivary gland and gastric epithelium. These tissues were excluded from SegPath.

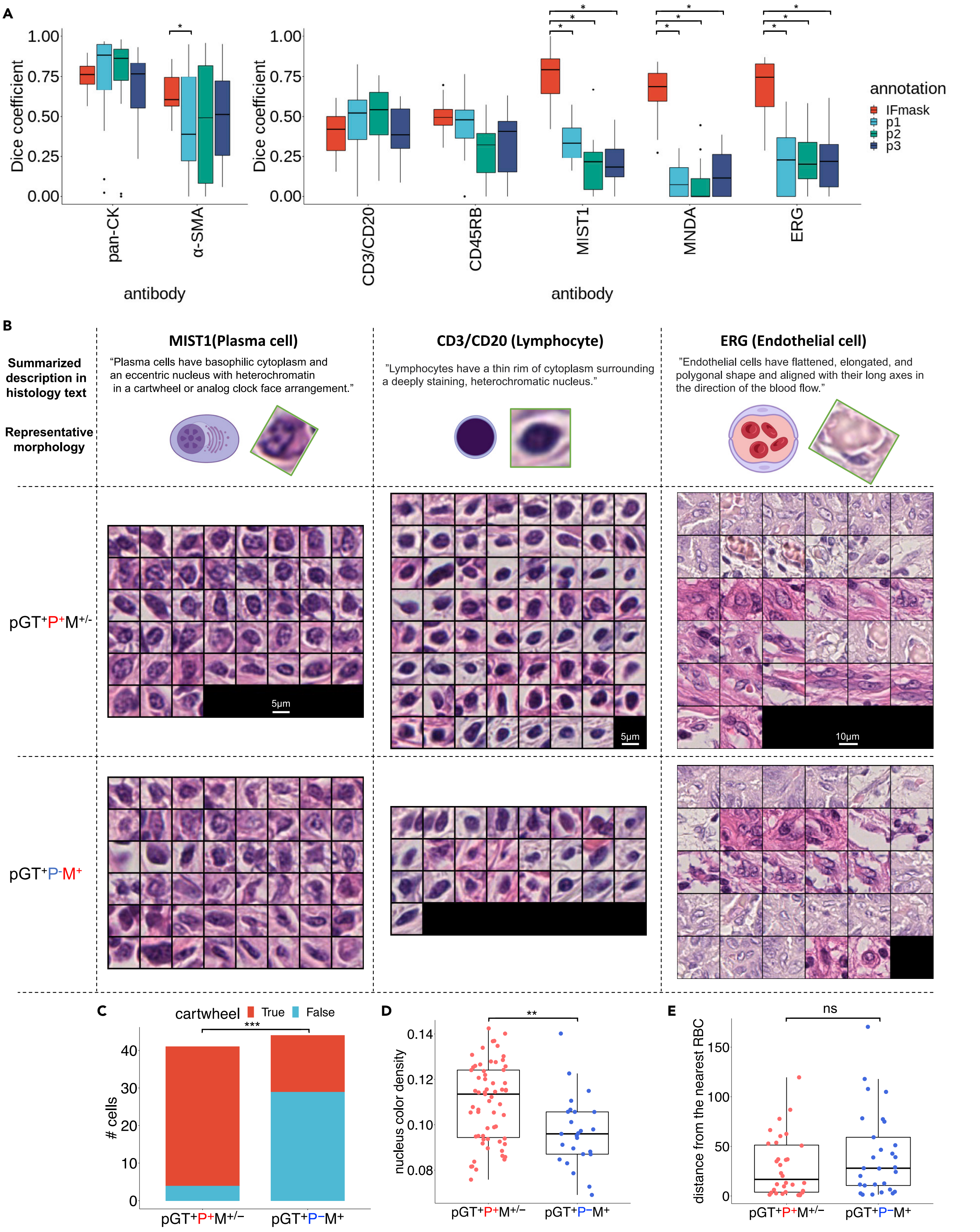

Figure 4. Evaluation of the annotation accuracy of SegPath

(A) Annotation accuracy of pathologists and the IF-masks in SegPath (n = 20 patches of 217.5 × 217.5 μm for each tissue or cell type) compared with pGT as ground truth. Dice coefficients of the IF-masks were compared with those of annotations by each pathologist. Two-sided Wilcoxon signed-rank test was used,

(legend continued on next page)

(MIST1), myeloid (MNDA), and endothelial (ERG) cells. Unlike the HE-paths, the performance of leukocytes (CD45RB) and lymphocytes (CD3/CD20) was lower than that of the other three cell types. This may have been because of the antibodies used to recognize the proteins in the cell membrane. This complicated the estimation of the exact locations of the cells, particularly with the variable intensity of the staining. Nevertheless, the performance of leukocytes and lymphocytes was comparable with that of the HE-paths.

We hypothesized that pathologists could not accurately identify cells with atypical morphologies. To clarify the biases in the annotations of the pathologists, we analyzed images of cells that the pathologists correctly identified ($pGT^{+}P^{+}M^{+-}$) and those that the pathologists could not correctly identify but that the IF-masks could identify ($pGT^{+}P^{-}M^{+}$) (Figures 4B and S6). Although the pathologists may have overlooked some cells, the morphological characteristics of the cells that the pathologists correctly identified were clarified. Overall, the shapes and sizes of $pGT^{+}P^{+}M^{+-}$ cells were more uniform than those of $pGT^{+}P^{-}M^{+}$ cells, implying a bias in the decisions of the pathologists toward the typical morphologies. Furthermore, we quantitatively investigated the bias of $pGT^{+}P^{+}M^{+-}$ morphology toward textbook descriptions. For example, plasma cells generally have a basophilic cytoplasm and an eccentric nucleus with heterochromatin in a characteristic cartwheel or clock-face arrangement. As expected, the plasma cells in $pGT^{+}P^{+}M^{+-}$ tended to have cartwheel-shaped nuclei (Figure 4C) but less so in $pGT^{+}P^{-}M^{+}$ cells, suggesting that pathologists cannot accurately identify plasma cells without clear cartwheel-shaped nuclei. Conversely, the basophilicity of the cytoplasm and eccentricity of the nucleus were not significantly different between $pGT^{+}P^{+}M^{+-}$ and $pGT^{+}P^{-}M^{+}$ cells (data not shown). Lymphocytes are generally characterized by a high nuclear/cytoplasmic ratio and dense nuclei. However, the lymphocytes overlooked by the pathologists often had thinner nuclei (Figures 4D and S7). There were no significant differences in the shapes of the vascular endothelial cells, but they were more likely to be correctly identified if they were surrounded by multiple RBCs (Figure 4E). With the myeloid cells, the pathologists were unlikely to miss polymorphonuclear leukocytes, such as neutrophils, as they are easy to identify (Figure S6).

The morphologies of cells that presented false negatives in the IF-masks but true positives in the HE-paths ($pGT^{+}P^{+}M^{-}$) were also examined (Figure S8). The results showed that most of the false negatives were due to the lack of false negatives for cell nuclei detection by Cellpose. The reason underlying this is unclear, but it may be due to the accuracy of the deep-learning model used in Cellpose. However, morphological bias was unclear on visual inspection.

In summary, we revealed an inherent morphological bias in the annotations of pathologists. However, the SegPath annotations are likely to be less prone to such bias and may enable the production of accurate segmentation models to cover the morphological diversity of cells.

Segmentation model trained on the dataset

We generated numerous annotated histological images of various tissues or cell types with diverse morphologies. To investigate whether such large-scale datasets improve segmentation performance, we trained semantic segmentation models on the part of the training set of SegPath for each cell type independently using a convolutional neural network (see "training deep neural network for segmentation" for the detailed procedure). We selected training patches randomly from the training dataset for each tissue/cell type until the number of patches or cells reached the target number (Table S6); this process was repeated three times for each target number. We evaluated the segmentation performance gains for the test set as a function of increasing patches for epithelia, smooth muscle cells/myofibroblasts, and the number of endothelial cells, leukocytes, lymphocytes, plasma cells, and myeloid cells (Figure 5). Similar to other image classification tasks for pathological images,[24] the predictive performance increased as more samples were used for model training. Apart from RBCs (CD235a), the performance gain did not seem to be saturated, indicating that more annotations can improve the segmentation performance. This result indicates the importance of our approach in obtaining a large number of annotated images in a high-throughput manner with minimal pathologist intervention.

We then evaluated the performance of the segmentation models trained using the entire training data (Figure 6A) in SegPath and tested it on the same part of the test dataset, as described above. We observed that the overall performance of the segmentation models was comparable with that of the pathologists (HE-path) assessing the epithelia, smooth muscles, leukocytes, and lymphocytes, and, surprisingly, more optimized than that of the pathologists assessing the other tissues or cell types in terms of the Dice coefficient. Cells that were not identified by the pathologists but identified by the trained models are

and p values were adjusted using the Benjamini-Hochberg method. $p < 0.05$ was considered statistically significant, as shown by asterisks. See also Figures S4 and S5; Table S5.

(B) Ground truth (pGT) cell images annotated by multiple pathologists ($pGT^{+}P^{+}M^{+-}$) and not identified by multiple pathologists but successfully annotated by the masks ($pGT^{+}P^{-}M^{+}$) in the ten patches. The illustrations and the actual images of the representative cell morphologies and sentences describing the morphologies written in a histology textbook are shown in each cell type.[23] Original illustrations from BioRender were used except for the lymphocyte, whose nucleus was denser and larger than the original illustration. The image was adjusted to be more similar to the representative morphology. For the box plot, the lower and upper hinges correspond to the 25th and 75th percentiles, respectively; the upper whisker extends from the hinge to the largest value no further than 1.5× interquartile range (IQR) from the hinge. The lower whisker extends from the hinge to the smallest value at 1.5× IQR of the hinge. pGT, ground truth; P, HE-path; M, IF-mask. See also Figure S6.

(C) Distribution of plasma cells with or without the typical cartwheel-shaped nuclei (n = 41 cells for $pGT^{+}P^{+}M^{+-}$ and n = 44 cells for $pGT^{+}P^{-}M^{+}$, two-sided Fisher's exact test).

(D) Nucleus hematoxylin intensity of lymphocytes (n = 63 cells for $pGT^{+}P^{+}M^{+-}$ and n = 25 cells for $pGT^{+}P^{-}M^{+}$, two-sided Mann-Whitney U test). See also Figure S7.

(E) Distance (μm) from the endothelial cell to the closest RBC (n = 32 cells for $pGT^{+}P^{+}M^{+-}$ and n = 29 cells for $pGT^{+}P^{-}M^{+}$). ***$p < 0.0001$, **$p < 0.01$. See also Figure S8.

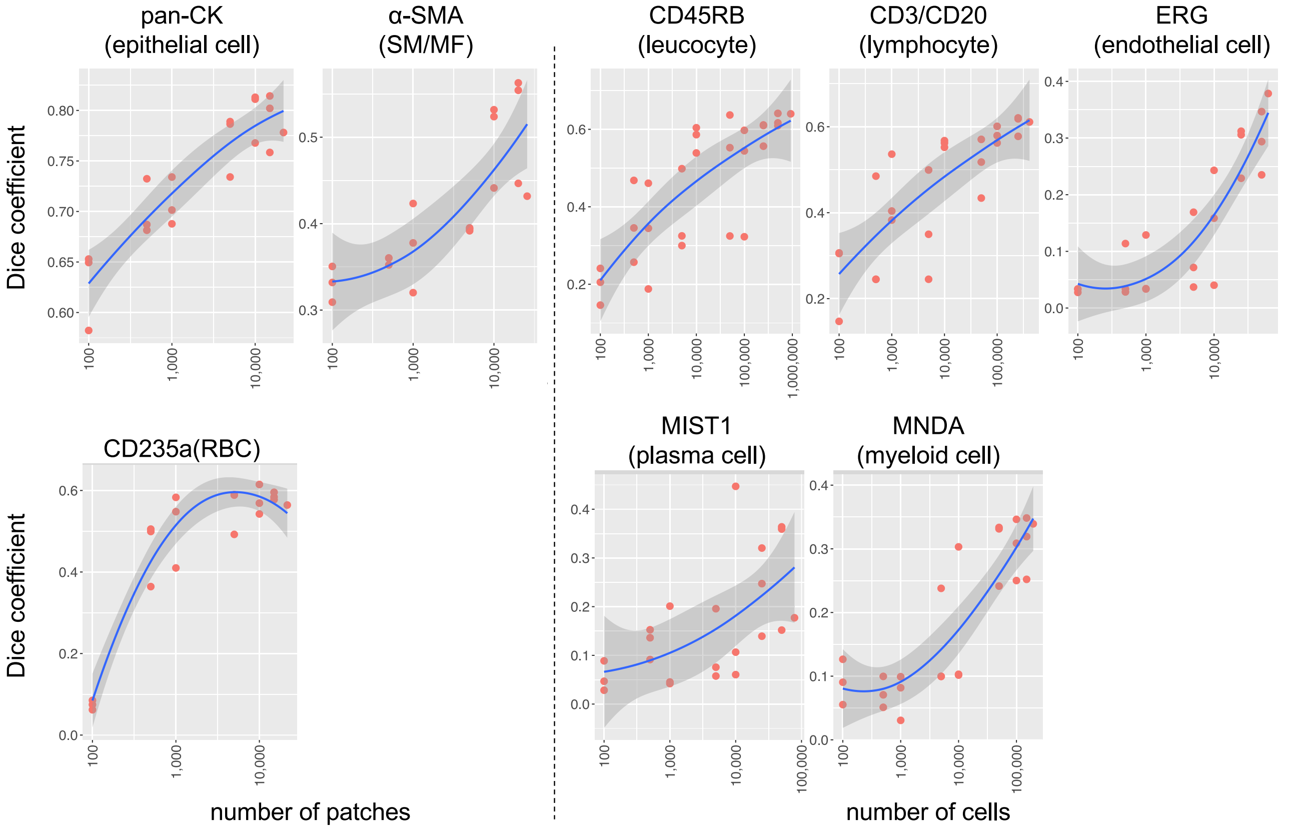

Figure 5. Effect of training sample sizes on segmentation models

Each point represents the Dice coefficient (F1 score) of the segmentation model trained on a randomly selected training dataset. The test dataset is the same for each tissue/cell type. The lowess smoothed curve with its 95% confidence interval is also shown in each plot. SM, smooth muscle; MF, myofibroblast. See also Table S6.

shown in Figure S9. Similar to the IF-masks, plasma cells without typical cartwheel-shaped nuclei (Figure 6B) and lymphocytes with thin nuclei were detected using the segmentation models more often than by the HE-paths (Figures 6C and S10). These results indicate that the datasets enable the segmentation models to cover diverse morphologies. As shown in the epithelial cells in Figure 6D, the segmentation models could identify even small areas that are difficult to discern. These results may be useful in cases of solitary cancer cells, such as those in diffuse-type gastric cancer (Figure S11). As shown in Figure 6D, the segmentation models were able to identify smooth muscle around blood vessels, which is normally difficult to identify, possibly owing to the lack of clear boundaries within the surrounding tissue.

To assess the generalization performance of the models trained on the SegPath dataset, we subsequently applied the models to the two external datasets, GlaS for the epithelium segmentation and NuCLS for the lymphocyte segmentation, without any training on the datasets (Figure 6E). Because ground truths in the datasets were generated by the pathologist solely based on the H&E images, we selected these two cell types of the high concordance between the pGT and the pathologists in our previous experiments (Figure 4A). The GlaS dataset was scanned using a Zeiss MIRAX MIDI Slide Scanner with a pixel resolution of 0.465 μm, and the NuCLS dataset was scanned using an Aperio Scanner with a pixel resolution of 0.20 μm, both of which differ from the SegPath dataset. The segmentation results show accurate segmentation despite the models having been trained only on the SegPath dataset, and the scanning conditions being different from those of SegPath (Dice coefficient: GlaS 0.681 ± 0.169; NuCLS 0.646 ± 0.320). The results demonstrate the generalization potential of the SegPath dataset.

Finally, the segmentation models were applied to external gigapixel WSIs from various cancer tissues (Figures S12–S15). To combine the outputs of the segmentation models for each tissue or cell type to generate a multi-tissue/cell segmentation result, we utilized the cell lineage hierarchy (see experimental procedures for details), such that leukocytes included lymphocytes, myeloid cells, and plasma cells, and the tissue regions not positive by any models were labeled "stroma." Therefore, we generated segmentation results for nine tissues and cell types. The densities of the predicted smooth muscle/myofibroblast regions varied; perivascular smooth muscle cells were dense, but other stromal regions were less dense, as shown in Figure S12. This reflected the expression of αSMA in smooth muscle cells or CAFs with myofibroblast phenotypes as discussed above. Although there is no ground truth for the dataset, the pathologist verified that the models were likely to capture the characteristic structures of various tumors, including benign and malignant

CellPress
OPEN ACCESS

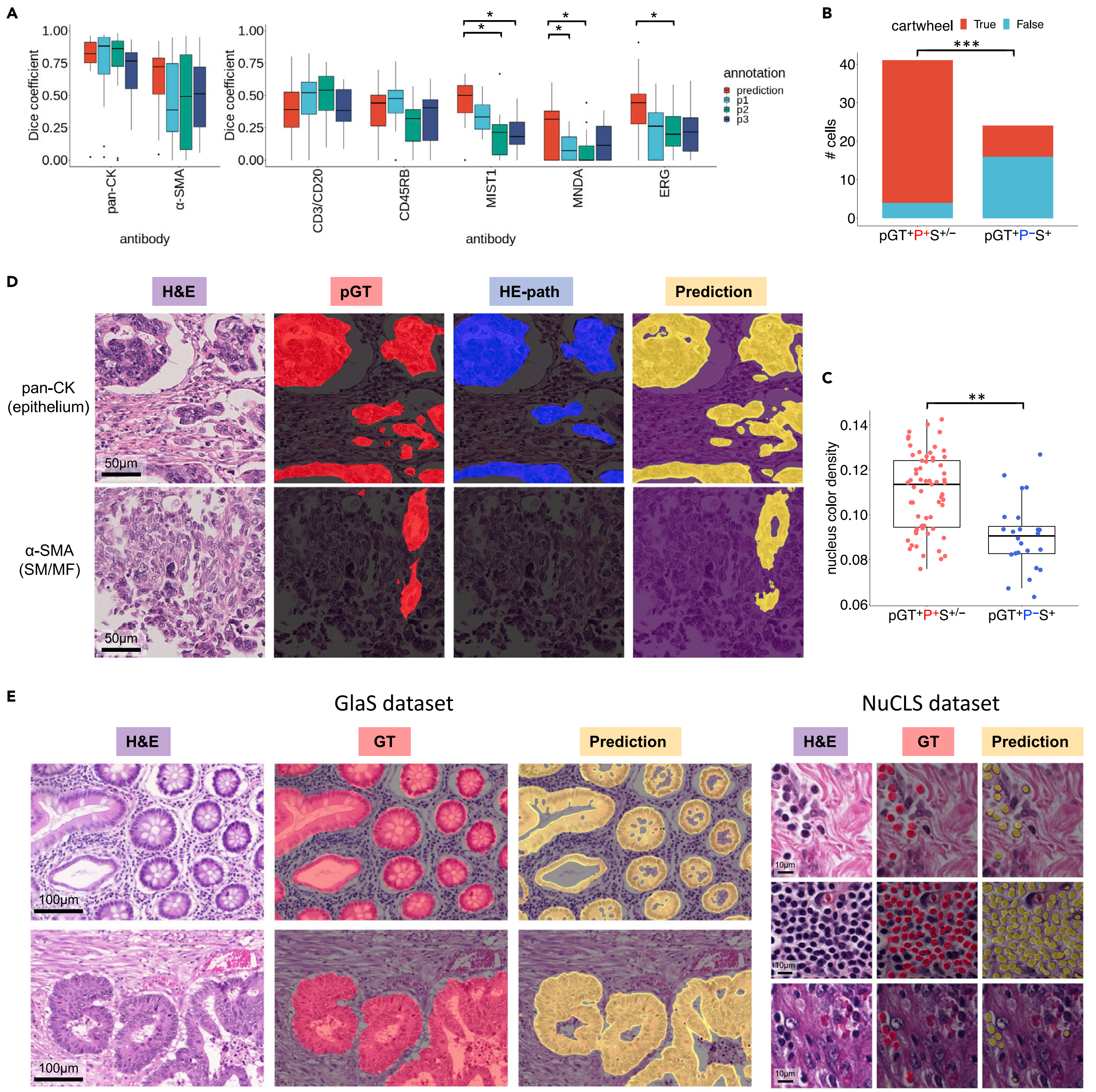

Figure 6. Performance evaluation of the segmentation models trained on the generated annotation masks

(A) Comparison of the annotation accuracies between pathologists and prediction of the segmentation models in terms of the Dice coefficient (F1 score) (n = 10 patches of 217.5 × 217.5 μm for each tissue or cell type). The optimal segmentation model in terms of validation loss was applied, and each point in the box plots represents a patch. pGT annotations were made by pathologists who evaluated both H&E and the corresponding IF images. Regions or cells annotated by at least two of the three pathologists were used. $p < 0.05$ was considered statistically significant, as shown by asterisks. See also Figure S9 and Table S5.

(B) Distribution of plasma cells with or without typical cartwheel-shaped nuclei (n = 41 cells for pGT$^+$P$^+$S$^{+/-}$ and n = 27 cells for pGT$^+$P$^-$S$^+$).

(C) Nuclear hematoxylin intensity of lymphocytes (n = 63 cells for pGT$^+$P$^+$S$^{+/-}$ and n = 24 cells for pGT$^+$P$^-$S$^+$). For the box plot, the lower and upper hinges correspond to the 25th and 75th percentiles, respectively; the upper whisker extends from the hinge to the largest value no further than 1.5× IQR from the hinge. The lower whisker extends from the hinge to the smallest value at 1.5× IQR of the hinge. pGT, ground truth; P, HE-path; S, prediction by the segmentation model. ***$p < 0.0001$, **$p < 0.01$. See also Figure S10.

(D) Comparison of pathologist annotations for AE1/3 and SMA.

(E) Samples of the segmentation results based on GlaS and NuCLS for epithelium and lymphocytes, respectively. Note that the segmentation models were trained using only the SegPath dataset, and no fine-tuning was performed on the target dataset. See also Figures S11–S15.

salivary gland tumors, which were not included in the training data. For example, lymphoid structures filled with many lymphocytes, vascular wall linings with endothelial cells surrounded by smooth muscle cells and containing RBCs, and the rich infiltration of plasma cells around cancer cells were detected (Figure S12). Additionally, the infiltration of small cancer foci with no apparent glandular formation was successfully identified in the gastric cancer specimen (Figure S13A). A dense lymphoid stroma and double layer of oncocytic epithelia were also successfully identified in the Warthin's tumor specimen (Figure S15A).

DISCUSSION

Owing to its simplicity and accuracy when appropriate antibodies are used, pathologists and biological researchers have routinely used IHC to identify specific cells or tissues in research and clinical practice. This study resolved the problem of annotation generation for tissue or cell segmentation by leveraging immunostaining with cell-specific or tissue-specific antibodies. We generated SegPath, an accurate and high-volume dataset for the tissue or cell segmentation of H&E images based on a workflow that utilizes IF staining. In SegPath, we targeted eight cell or tissue types that constitute the major component in the tumor microenvironment,[25] and the granularity in the cell hierarchy was based on the potential feasibility of segmentation in H&E images. The advantages of our workflow were that identical sections were stained with H&E and IF, which enabled the precise localization of target tissues or cell types. Furthermore, higher annotation accuracy could be achieved even if the target tissues or cells presented atypical morphologies. A series of experiments showed that the generated masks and segmentation models trained on the dataset achieved good performance with various morphologies. Although each image contained a mask for only one tissue or cell type, multiple cell types or tissues may be segmented using the outputs from multiple models, as shown in the last experiment.

Our experiment revealed that pathologists could miss or mark incorrect labels with variable extents depending on the cell type. Furthermore, the annotations of the pathologists were biased toward typical morphologies. Cells with atypical morphologies and/or surrounding microenvironments may be subtypes with unique functions or states, as suggested by previous studies.[26,27] The datasets of existing studies based on annotations by pathologists also contained biases toward typical morphologies; therefore, the model trained on the training dataset had the same inherent bias. Our workflow was able to resolve these problems; therefore, the model trained on the dataset can yield more accurate characterization of tumor tissues.

We further showed that the annotation accuracy increased as the number of annotated cells increased. In most cell types the accuracy did not saturate, even with a large number of annotations in SegPath. This may be because cell morphology is more diverse than what is currently known, and our dataset comprehensively covers diversity. Another possibility is that the segmentation models had a receptive field that exceeded the range of cells; therefore, it considered the surrounding environment to make comprehensive judgment. Hence, it is important to create datasets with various tissues and specimens, which was an advantage of our approach when using immunostaining TMAs.

There are other experimental methods for identifying multiple cell types simultaneously in a tissue section, such as highly multiplexed IF,[28] imaging mass spectrometry,[29] and spatial transcriptomics.[30] Such methods are more accurate than our approach and can identify cells that cannot be detected in H&E-stained sections. However, these methods are costly, labor intensive, and require additional equipment and experiments, including the optimization of experimental conditions.[31] Segmentation from H&E-stained tissues is a complementary approach to such methods because it does not require additional equipment. More importantly, H&E staining accounts for approximately 80% of all human tissue staining performed globally,[32] and the method can be applied to the large number of specimens accumulated thus far. Additionally, a high-throughput analysis that will allow simultaneous comparison of multiple samples can be achieved by applying TMAs to glass slides containing fragments of tissues from numerous patients. Such advantages enable comprehensive pathomics, which can be used to analyze the correlation between cell or tissue distribution and clinical information such as genomics data.[33]

We have made this large-scale dataset accessible to the public to enhance pathology-based cancer research and segmentation algorithm development. We plan to expand the datasets to include more cell types and facilitate finer segmentation. Our approach will enhance high-throughput computational pathology by adding information, such as the tissue context, rather than the image level category, and could lead to improved diagnostic techniques and drug development for cancer patients.

Limitations of the study

This study was limited by various errors and inconsistencies in the dataset owing to uneven IF staining, non-specific staining, and errors in the cell recognition model. However, according to a previous study,[34] the supervised segmentation method is sensitive to biased errors and robust to unbiased errors. The dataset generated in our workflow is less biased than those generated by pathologists in terms of morphology. Our results showed that the model trained on our dataset outperformed the assessments made by pathologists of several cell types, suggesting that the model can detect cells with atypical morphologies. Additionally, emerging techniques for robust learning under random label noise, such as constrained reweighting, can be used to develop more accurate segmentation models.[35,36] Another limitation of the study is that the cells with atypical morphology could be overlooked by the deep-learning model during the training process if they are very rare. However, dedicated training techniques, such as hard sample mining, could resolve this problem.

EXPERIMENTAL PROCEDURES

Resource availability

Lead contact

Further information and requests for resources should be directed to and will be fulfilled by the lead contact, Shumpei Ishikawa (ishum-prm@m.u-tokyo.ac.jp).

Materials availability

This study did not generate new unique reagents.

Data and code availability

SegPath datasets for each antibody have been deposited in Zenodo and are publicly available as of the date of publication. The links to the Zenodo repository are summarized at https://dakomura.github.io/SegPath.

All original codes for the generation of SegPath have been deposited at github under https://doi.org/10.5281/zenodo.7502875 and are publicly available as of the date of publication.

Any additional information required to reanalyze the data reported in this paper is available from the lead contact upon request.

Sample preparation and image data acquisition

All histopathological specimens used in the generation of SegPath were obtained from patients who were diagnosed between 1955 and 2018 and had undergone surgery at the University of Tokyo Hospital. TMAs for various cancers (including glioma, meningioma, ependymoma, kidney renal clear cell carcinoma, lung adenocarcinoma, lung squamous cell carcinoma, breast adenocarcinoma, gastric adenocarcinoma, colon adenocarcinoma, pancreatic adenocarcinoma, cholangiocarcinoma, hepatocellular carcinoma, esophageal squamous cell carcinoma, head and neck squamous cell carcinoma, urothelial tumors, bladder cancer, prostate adenocarcinoma, sarcoma, melanoma, uterine cancer, ovarian tumors, and testicular germ cell tumors) were constructed from the FFPE tissue blocks used for pathological diagnoses. Two TMA spots for each patient were included in each TMA block. The TMA FFPE blocks were cut to obtain 3-μm-thick sections. All histopathological specimens were anonymized in an unlinkable manner; therefore, the requirement for informed consent was waived. This study was approved by the Institutional Review Board of the University of Tokyo. Information on the histopathological specimens is summarized in Table S2.

To create the SegPath dataset, we obtained histopathological images of both H&E- and IF-stained sections from the same TMAs as follows. For H&E staining, the sections were deparaffinized and rehydrated by immersion in xylene (#241-00091, FUJIFILM Wako Pure Chemical, Osaka, Japan) and ethanol (#057-00451, FUJIFILM Wako Pure Chemical), respectively. Hematoxylin (#6187-4P, Sakura Finetek Japan, Tokyo, Japan) and eosin (#8660, Sakura Finetek Japan) solutions were used for H&E staining following the manufacturer's protocols. The stained sections were dehydrated by immersion in ethanol followed by xylene. Glass coverslips (Matsunami Glass, Osaka, Japan) with Marinol (#4197193, Muto Pure Chemicals, Tokyo, Japan) were used to cover the stained sections. H&E staining, using the same protocol, was also performed to create WSIs for evaluating multi-cell-type segmentation among resected specimen sections. WSIs of the H&E-stained sections were captured using a Hamamatsu Nanozoomer S60 whole-slide scanner (Hamamatsu Photonics, Shizuoka, Japan) at 40× (0.220818 μm/pixel) resolution. Next, we used the same sections of H&E-stained TMA sections for IF. The glass coverslips were removed by immersing the slides in xylene, rehydrating with ethanol, and washing with distilled water. For the destaining of H&E and antigen retrieval, the slides were autoclaved for 5 min at 120°C and immersed in citrate buffer (pH 6.0) (Abcam, Cambridge, UK). Endogenous peroxidase activity was measured using 0.3% hydrogen peroxide (Sigma-Aldrich, St. Louis, MO, USA) in methanol (#137-01823, FUJIFILM Wako Pure Chemical) for 15 min, followed by washing with distilled water. Non-specific protein-protein reactions were blocked by incubating the sections in Antibody Diluent/Block (#ARD1001EA, PerkinElmer, Waltham, MA, USA) for 15 min at room temperature. The following primary antibodies were used, as summarized in Table 1: monoclonal mouse immunoglobulin G (IgG) anti-pan-cytokeratin, clone AE1/AE3 (without dilution; IS05330-2J; DAKO, Carpinteria, CA, USA); monoclonal mouse IgG anti-human αSMA, clone 1A4 (1:200 dilution; M085129-2; DAKO); monoclonal mouse IgG anti-human CD45RB, leukocyte common antigen, clones 2B11 + PD7/26 (1:200 dilution; IR75161-2J; DAKO); monoclonal mouse IgG anti-human N-terminal ERG, clone 9FY (without dilution; PM421AA; Biocare Medical, Concord, CA, USA); monoclonal mouse IgG anti-glycophorin A, clone JC159 (1:200 dilution; MA5-12484; Thermo Fisher Scientific, Waltham, MA, USA); polyclonal rabbit anti-human MNDA (1:1,000 dilution; HPA034532-100UL; Sigma-Aldrich); monoclonal rabbit IgG anti-human MIST1/bHLHa15 protein, clone D7N4B (1:100 dilution; #14896; Cell Signaling Technology, Beverly, MA, USA); polyclonal mouse anti-human CD3 (1:200 dilution; IS50330-2J; DAKO); and monoclonal mouse IgG anti-human CD20cy, clone L26 (1:200 dilution; IS60430-2J; DAKO). IF staining using each of the aforementioned primary antibodies (AE1/AE3, αSMA, CD45, ERG, glycophorin A, MNDA, MIST1, and CD3/CD20 mix) was performed for 2 h at 4°C, according to the instructions of Opal Multiplex IHC Kit (#NEL811001KT; PerkinElmer). Opal Polymer HRP solution (ARH1001EA; PerkinElmer) was used to enhance the signals by incubating the sections for 10 min at room temperature. The sections were then incubated with 100 μL of Opal 690 fluorophore (1:10 dilution; FP1497001KT; PerkinElmer) at room temperature for 10 min to achieve 690-nm single-color IF staining. Nuclear staining was performed with DAPI solution (FP1490A; PerkinElmer) at room temperature for 5 min. The slides were then covered with glass coverslips (Matsunami Glass) using Prolong Gold antifade reagent with DAPI (P36931, Thermo Fisher Scientific). WSIs of the IF staining TMA slides were captured using a Hamamatsu Nanozoomer S60 whole-slide scanner at 40× (0.220818 μm/pixel) resolution.

Whole-slide image pre-processing

Large artifacts (i.e., tissue folds and air bubbles) in each WSI were marked by pathologists before analysis. In the patch-extraction process, patches overlapping the marked regions or heavily blurred regions with a variance of the Laplacian filter[37] <0.0005 in the grayscale image were removed. Additionally, tissue region candidates were extracted from grayscale H&E slides at zoom level 4 (1/16 of the 40× resolution) by applying Otsu binarization after Gaussian blur with an 81 × 81-kernel. Connected regions ranging from 12.8 to 256 million pixels^2 in size at 40× resolution were regarded as the tissue regions. After the rigid registration described below, patches within the tissue regions of 1,024 × 1,024 pixels with a stride of 1,024 pixels were then extracted. The patches within 200 pixels at 40× resolution from the edges of the tissue regions were discarded because non-specific IF staining is often observed at the edge of the tissue.[38]

Image registration and patch extraction

To create masks for the deep-learning model of H&E-stained histological images, each IF image was registered to the H&E-stained image of the same slide. Image registration was performed using a multi-step procedure that began with coarse WSI-level registration and proceeded to fined-grained, patch-level registration. Nuclear regions were considered in the calculation to accurately align the two images. Specifically, the hematoxylin color component extracted using the scikit-image's "rgb2hed" function in the H&E image and DAPI channel component in the IF image were used for registration. First, discrete Fourier transform (DFT)-based rigid registration was performed to estimate the optimal vertical and horizontal translation between H&E WSI and paired IF WSI at zoom level 6 (1/64 of the 40× resolution). After the patch pairs of 1,024 × 1,024 pixels at zoom level 1 (1/2 of the 40× resolution) had been extracted from the same position of the aligned WSI pairs, DFT-based rigid registration was performed again to obtain a finer-grained registration, and the vertical and horizontal translation levels were recorded. Kernel density estimation using Gaussian kernels was applied to the two-dimensional distribution of the translations, and the vertical and horizontal translation levels with the highest densities were used to register all image pairs in the same WSI. Subsequently, 1,024 × 1,024-pixel tiles at 40× resolution were extracted again from the aligned WSI pairs. After two additional rounds of the same DFT-based rigid registrations at zoom levels 1 and 0 at 40× resolution, non-rigid registration using the Demons algorithm[39] was applied after the histogram matching filter. We used a multi-resolution pyramid with three layers (with shrinkage factors of 8, 4, and 2 and a smoothing sigma of 12, 8, and 4). A gradient descent with a learning rate of 1.0 and 20 iterations was used for parameter optimization. Finally, 20-pixel margins from the edges were removed, such that the image did not include unregistered regions.

Initial mask generation

For mask generation, it is necessary to determine the cut-off values for positive IF signals and remove false-positive signals due to artifacts, registration errors, or non-specific signals from blood cells.

Inconsistencies between the intensities of the DAPI nuclear channel in the IF image and the hematoxylin component in the H&E-stained image, indicating the existence of artifacts or registration errors, were detected by calculating the Pearson's correlation coefficient between the two signal intensities. Patches with correlation coefficients below 0.5 were removed for further analysis. False-positive signals derived from the autofluorescence of RBCs were

removed by masking the positively predicted regions using the RBC segmentation neural network trained on the anti-CD235a antibody-stained dataset. Based on visual inspection, an IF signal intensity >50 (epithelium, smooth muscle, and RBCs) or 25 (others) was regarded as a positive signal in the initial mask generation step.

For the epithelium and smooth muscle, the positive signal area was used as a segmentation mask without modification. For RBCs, the area that was positive in the IF image and red in the H&E-stained image (R > 100 and G < 130, and R > B) was used as a segmentation mask. For leukocytes, myeloid cells, lymphocytes, plasma cells, and endothelial cells, positive signals from the target cells were transferred into the nuclei based on the IF staining pattern to obtain a more consistent result and improve the interpretability of the segmentation model. Cellpose version 0.6.5[19] was applied to the DAPI nuclear channel in the IF images to detect the nuclei. We selected a model with the following parameters: diameter = 30, channels = [3,0], batch_size = 64, and cellprob_threshold = 0.1. Nuclei were masked if over 40% of them contained positive signals. Finally, one iteration of morphological erosion with a 3 × 3 kernel was applied to each region of the nuclei to prevent multiple cells from sticking together, which could cause an underestimation of the cell count.

For deep neural network training during the mask generation process, all patches were divided into training, validation, or test sets so that all patches from the same TMA spot belonged to the same set. TMA spots in each TMA were detected as clusters by applying the DBSCAN clustering algorithm[40] implemented in scikit-learn to patches using the x and y coordinates as the input features, maximum distance set to 3,000 pixels, and min_samples set to 5. The validation and test sets contained patches from two TMA spots in each TMA slide, and the rest were placed into the training set. For deep neural network training after mask generation, we moved the training/validation patches from the patient in the test set to the test set and training patches from the patients in the validation set to the validation set, so that the patches from the same patient did not span the training/validation/test sets.

Training deep neural network for segmentation

The encoder-decoder neural networks were trained for semantic segmentation. The combination of the encoder and decoder was independently optimized for each cell type or tissue. The backbone of the encoder was a pre-trained convolutional neural network, such as ResNet[41] trained on the 2012 ILSVRC ImageNet dataset, or EfficientNet[42] trained on 300 million unlabeled images from JFT[43] using noisy student training.[44] The decoder module was selected from one of three models: U-net,[45] U-net++,[46] or DeepLabV3+.[47] The network was trained using randomly sampled patches with sizes of 984 × 984 pixels and batch sizes of 16. During training, the weights in all layers of the decoders and segmentation head were updated through the RAdam optimizer with a weight decay of 1×10^{-4}, $\beta_1 = 0.9$, and $\beta_2 = 0.999$. Data augmentation and normalization were applied in the following order:

- Random crop to 640 × 640 pixels
- Color, contrast, and brightness augmentation (hue [−0.1, 0.1], saturation [0.9, 1.1], contrast [0.9, 1.1], and brightness [0.9, 1.1])

The data were normalized to mean = [124.0, 116.0, 104.0] and SD = [58.6, 57.3, 57.6].

- Random horizontal and vertical flips
- Random affine transformation with rotation with up to 180°, and scale with scaling factor ranging from 0.9 to 1.1 with reflection padding
- Random Gaussian blur of a 3 × 3 kernel with probability = 0.3

The backbone and architecture of the deep-learning model and hyperparameters, including the learning rate, were optimized using the tree-structured Parzen estimator algorithm[48] based on the validation Dice score. The validation Dice score was evaluated across all images at once instead of averaging the Dice scores of each patch, as the positively stained areas varied drastically among patches. The hyperparameters optimized in this study are listed in Table S4. All segmentation models were trained for 25 epochs. At least five trials were tested, and the model with the optimal validation Dice score was selected for the subsequent analysis. The model architecture and decoder in the final trial are shown in Table S5.

Improvement of cut-off intensity and nucleus overlap ratio

The cut-off values of the signal intensities (= 25 or 50) and nucleus overlap rate (40%) in the initial mask generation may not be optimal. Because we observed heterogeneity in the signal intensities of some of the sections, a single cut-off value for the signal intensity across one TMA slide may not be appropriate. Otsu's banalization is often applied in similar scenarios, but it is difficult to differentiate patches with overall low signals because of weak staining of positive cells or the absence of positive cells, and the latter results in many false-positive masks.

We observed that the staining strength gradually changed in the section. The segmentation network could detect positive cells with a certain level of accuracy, even if it was trained on the initial mask. Based on this observation, we iteratively improved the cut-off values. First, linear ridge regression analysis was carried out to detect patches with positive cells by setting the intercept to zero, where the explanatory value was the IF intensity. The dependent variable was the cell probability of the trained deep neural network model, both of which were smoothed by Gaussian blur with an 11 × 11 kernel. Patches with a regression coefficient >1 and maximum IF intensity >10 without RBC regions were considered positive. For each positive patch, the initial cut-off values were determined by applying Otsu's binarization to the patch and the nearest eight positive patches. To avoid extreme cut-off values, they were clipped to a minimum of 10 and a maximum of 50. For the epithelia, the cut-off was reduced by 20% as we observed the heterogeneous staining of anti-pan-CK antibodies between the cytoplasm and nuclei, with weaker signal intensities in the nucleus. Finally, the thresholds for each patch, including the negative patch, were determined using the weighted average cut-off values of the nearest 16 positive patches. A Gaussian distance weight of 1/3,000 pixel from the target patch was used for the weight. For leukocytes, myeloid cells, lymphocytes, plasma cells, and endothelial cells, the nucleus overlap rate cut-off, which maximizes Matthew's correlation coefficient between the prediction and mask within the range of 10%–80%, was used. In contrast to the signal intensity cut-off, the same cut-off value was adopted for each cell type. Based on the new segmentation masks, the segmentation networks were trained again, and the cut-off intensity and nucleus overlap ratio were optimized. These processes were repeated twice to verify that the mask remained almost unchanged after the second optimization.

Annotation by pathologists

For each cell type, except RBCs, ten patches were randomly selected from the training data. Three trained pathologists independently performed the annotation task for the patches using the Labelbox annotation tool (Labelbox, San Francisco, CA, USA). Tissue regions were selected with polygonal annotations, whereas cells were selected with point annotations to the center of the nuclei. In the first round, only H&E images were shown to the pathologists and annotated. Regions or cells selected by at least two pathologists were used as the HE-path for subsequent experiments. In the next round, both H&E and IF images without DAPI overlaid with H&E images were shown to the same pathologists and annotated again. Regions or cells selected by at least two pathologists were used as pGT data. For point annotations to the cells, annotations by two pathologists were regarded as overlapping if they were within an 8-pixel distance (= 1.77 μm).

Evaluation of masks and predictions

The accuracy of the annotations was evaluated based on the Dice coefficient between the pGT and HE-path, IF-mask, or prediction. The pixel- and cell-level Dice coefficients were calculated for tissue and cell segmentations, respectively. The HE-path and IF-mask or prediction were regarded as overlapping if any point in the HE-path was on the IF-mask or predicted region.

Morphological evaluations

We evaluated the morphological parameters of the annotated cells: cartwheel-shaped nuclei for plasma cells, nucleus density for lymphocytes, and distance from the nearest RBC to the endothelial cells. The presence of typical cartwheel-shaped nuclei in each plasma cell was determined by a pathologist. For lymphocyte intensity, the nucleus regions were annotated by a pathologist using Labelbox, and the mean intensity of the hematoxylin component estimated by the rgb2hed function in the scikit-image was

used for evaluation. For the distance from the nearest RBC to the endothelial cells, all RBCs were annotated by a pathologist using Labelbox, and the pixel distance between each endothelial cell and nearest RBC was used for evaluation.

Effect of training data size on the segmentation performance

Patches were individually sampled in the training set until the number of patches or cells reached the pre-determined value, as shown in Table S6. This process was repeated thrice for each value, except for the entire training set. Using these datasets, the deep neural network models were trained and tested on the same test set.

We trained a U-net with a resnet18 backbone pre-trained on ImageNet on the training dataset using the same procedure for all evaluation datasets. The network was trained using randomly sampled patches with a size of 960 × 960 pixels and batch size of 16. During training, the weights in all layers of the decoders and the segmentation head were updated using the RAdam optimizer with a learning rate of 0.01, weight decay of 1×10^{-4}, $\beta_1 = 0.9$, and $\beta_2 = 0.999$. The same augmentation and normalization were applied as described in the previous section. The Dice loss was used as the loss function. The model was trained for a maximum of 10,000 epochs. If the validation Dice coefficient did not increase for five consecutive epochs, early stopping was applied, and the optimal model based on the validation Dice coefficient was used for testing.

Validation cohort

To test the generalization performance of the SegPath dataset, we applied the models trained on the SegPath dataset to two external validation datasets: (1) GlaS dataset[8] for epithelium and (2) NuCLS dataset[7] for lymphocyte semantic segmentation. For GlaS, both training and test data, which consisted of 165 images from 74 benign and 91 malignant colon tissue images in total, were processed with the model with no training on the GlaS dataset. Because our semantic segmentation model for epithelia is not directly applicable for instant segmentation, individual gland information is not used in the evaluation. For the NuCLS dataset, a corrected single raster dataset, which consists of 452 patches with more than five lymphocytes from The Cancer Genome Atlas cases, was tested. For the GlaS dataset, Dice coefficient of the segmentation mask was used for the evaluation. In contrast, because NuCLS contains both segmentation mask for the segmentation and boundary box for detection, object-level Dice coefficient was used for the evaluation, where the object in the prediction is defined as the consecutive positive region, and any overlap between the contour and the segmentation mask or boundary box in the ground truth is considered as true positive. The only difference between image preprocessing and applying the models to the SegPath was scaling based on the mpp ratio (2.8 for GlaS and 0.9049 for NuCLS). The model ensemble approach for epithelium and lymphocytes using two or three different models with optimal validation Dice coefficients during training (Table S5) was used for the segmentation.

WSIs from two institutes were used for the validation of multi-cell-type segmentations of H&E-stained images. Specimens were obtained from (1) three patients with gastric adenocarcinoma who underwent surgery and were diagnosed at the University of Tokyo Hospital between 1955 and 2018 and (2) four patients with salivary gland tumors (salivary duct carcinoma, Warthin's tumor, and cystadenoma) who underwent surgery and were diagnosed at Tokyo Medical and Dental University Hospital between 1990 and 2020. Resected specimens of gastric adenocarcinoma were prepared from the FFPE blocks and sliced to a thickness of 6 μm. All histopathological specimens were anonymized. This study was approved by the Institutional Review Board of each university. Throughout these experiments, the multi-cell-type segmentation strategy described below was used.

Multi-cell-type segmentation

Based on the deep neural network model for the segmentation of each tissue or cell type, we performed multi-cell-type segmentations of the H&E-stained images. To consider the lineage hierarchy of cells or tissues, the segmentation results were merged, beginning with coarse categories and then overwritten with fine-grained categories. The four groups were defined as follows and overwritten in the order below. The following layer and label encoding were adopted:

[layer 0] 0: background; 1: stroma (other than smooth muscle cells)
[layer 1] 2: epithelial cells; 3: smooth muscle cells
[layer 2] 4: leukocytes; 5: endothelial cells; 6: red blood cells
[layer 3] 7: lymphocytes; 8: plasma cells; 9: myeloid cells

$x_{i,j}$, $c^k_{i,j}$, and $p^m_{i,j}$ denote the pixel intensity after grayscale conversion, predicted label at the kth layer, and output logit value of the segmentation model for m^{th} cell type at the $(i,j)^{th}$ pixel in the image, respectively.

$$c^0_{i,j} = \begin{cases} 1 & \text{if } x_{i,j} > t \\ 0 & \text{otherwise} \end{cases},$$

where t is Otsu's threshold for the WSI based on the pixel intensity after grayscale conversion. The predicted label was updated using the following recursive calculation:

$$c^k_{i,j} = \begin{cases} \underset{m \in M_k}{\operatorname{argmax}} p^m_{i,j} & \text{if } \underset{m \in M_k}{\max} p^m_{i,j} > 0 \\ c^{k-1}_{i,j} & \text{otherwise} \end{cases} \quad (k = 1 \ldots 3),$$

where M_k is the set of cell types in the kth layer and $c^3_{i,j}$ was used as the prediction label.

We applied the model ensemble approach for each tissue or cell type using 1–3 different models with optimal validation Dice coefficients during training (Table S5). To obtain the WSI-level segmentation results, inference was executed for a 7,680 × 7,680-pixel patch from the WSI, and the result of each patch was assembled into a WSI.

Implementation details

Rigid and non-rigid image registrations were performed using imreg version 2.0.1a (https://github.com/matejak/imreg_dft) and SimpleITK version 2.0.2 Python library, respectively. Kernel density estimation was performed using SciPy version 1.3.1. The neural networks were trained using Python version 3.8.5, PyTorch Lightning version 1.4.2 (https://www.pytorchlightning.ai/), and Segmentation Models PyTorch version 0.2.0 (https://github.com/qubvel/segmentation_models.pytorch). To speed up model training, mixed precision (16-bit) training implemented in PyTorch Lightning was used. Hyperparameter optimization was performed using Optuna version 2.7.0.[49] Training and testing were performed on the NVIDIA DGX-1 server with 8 NVIDIA Tesla V100 GPUs and 256 GB RAM.

SUPPLEMENTAL INFORMATION

Supplemental information can be found online at https://doi.org/10.1016/j.patter.2023.100688.

ACKNOWLEDGMENTS

We thank Shin Aoki for helping us stain tissue specimens. We thank Editage (https://www.editage.jp/) for the English language review. This study was supported by the AMED Practical Research for Innovative Cancer Control under grant number JP 22ck0106640 to S.I., the AMED Project Focused on Developing Key Technology for Discovering and Manufacturing Drugs for Next-Generation Treatment and Diagnosis under grant number JP 21ae0101074 to S.I., the AMED P-PROMOTE: Project for Promotion of Cancer Research and Therapeutic Evolution under grant number JP 22ama221502 to S.I., JSPS KAKENHI Grant-in-Aid for Scientific Research (S) under grant number 22H04990 to S.I., and JSPS KAKENHI Grant-in-Aid for Scientific Research (B) under grant number 21H03836 to D.K.

AUTHOR CONTRIBUTIONS

Conceptualization, S.I. and D.K.; methodology, D.K., K.S., and H.O.; software, D.K. and T.O.; validation, D.K. and T.O.; formal analysis, D.K.; investigation, D.K., T.O., and S.I.; resources, T.O. and T.U.; data curation, D.K., T.O., M.O., H.E., and H.K.; resources, T.I. and T.U.; annotation, M.H., M.O., and R.R.H.; writing – original draft, D.K., T.O., and S.I.; writing – review & editing, D.K. and S.I.; visualization, D.K.; supervision, S.I.

DECLARATION OF INTERESTS

The authors declare no competing interests.

Received: July 22, 2022
Revised: November 30, 2022
Accepted: January 12, 2023
Published: February 10, 2023

REFERENCES

1. Madabhushi, A. (2009). Digital pathology image analysis: opportunities and challenges. Imaging Med. *1*, 7–10.
2. Cooper, L.A., Demicco, E.G., Saltz, J.H., Powell, R.T., Rao, A., and Lazar, A.J. (2018). PanCancer insights from The Cancer Genome Atlas: the pathologist's perspective. J. Pathol. *244*, 512–524. https://doi.org/10.1002/path.5028.
3. Lal, S., Das, D., Alabhya, K., Kanfade, A., Kumar, A., and Kini, J. (2021). NucleiSegNet: Robust deep learning architecture for the nuclei segmentation of liver cancer histopathology images. Comput. Biol. Med. *128*, 104075. https://doi.org/10.1016/j.compbiomed.2020.104075.
4. Naylor, P., Laé, M., Reyal, F., and Walter, T. (2017). Nuclei segmentation in histopathology images using deep neural networks. In 2017 IEEE 14th International Symposium on Biomedical Imaging (ISBI 2017), pp. 933–936. https://doi.org/10.1109/ISBI.2017.7950669.
5. Graham, S., Jahanifar, M., Vu, Q.D., Hadjigeorghiou, G., Leech, T., Snead, D., Raza, S.E.A., Minhas, F., and Rajpoot, N. (2021). CoNIC: Colon Nuclei Identification and Counting Challenge 2022. Preprint at arXiv. https://doi.org/10.48550/arXiv.2111.14485.
6. Chen, H., Qi, X., Yu, L., and Heng, P.A. (2016). DCAN: Deep Contour-Aware Networks for Accurate Gland Segmentation. In 2016 IEEE Conference on Computer Vision and Pattern Recognition (CVPR), pp. 2487–2496. https://doi.org/10.1109/CVPR.2016.273.
7. Amgad, M., Atteya, L.A., Hussein, H., Mohammed, K.H., Hafiz, E., Elsebaie, M.A.T., Alhusseiny, A.M., AlMoslemany, M.A., Elmatboly, A.M., Pappalardo, P.A., et al. (2021). NuCLS: A scalable crowdsourcing, deep learning approach and dataset for nucleus classification, localization and segmentation. Preprint at arXiv. https://doi.org/10.48550/arXiv.2102.09099.
8. Sirinukunwattana, K., Pluim, J.P.W., Chen, H., Qi, X., Heng, P.-A., Guo, Y.B., Wang, L.Y., Matuszewski, B.J., Bruni, E., Sanchez, U., et al. (2017). Gland segmentation in colon histology images: The glas challenge contest. Med. Image Anal. *35*, 489–502. https://doi.org/10.1016/j.media.2016.08.008.
9. Amgad, M., Elfandy, H., Hussein, H., Atteya, L.A., Elsebaie, M.A.T., Abo Elnasr, L.S., Sakr, R.A., Salem, H.S.E., Ismail, A.F., Saad, A.M., et al. (2019). Structured crowdsourcing enables convolutional segmentation of histology images. Bioinformatics *35*, 3461–3467. https://doi.org/10.1093/bioinformatics/btz083.
10. Litjens, G., Bandi, P., Ehteshami Bejnordi, B., Geessink, O., Balkenhol, M., Bult, P., Halilovic, A., Hermsen, M., van de Loo, R., Vogels, R., et al. (2018). 1399 H&E-stained sentinel lymph node sections of breast cancer patients: the CAMELYON dataset. GigaScience *7*, giy065. https://doi.org/10.1093/gigascience/giy065.
11. Verma, R., Kumar, N., Patil, A., Kurian, N.C., Rane, S., Graham, S., Vu, Q.D., Zwager, M., Raza, S.E.A., Rajpoot, N., et al. (2021). MoNuSAC2020: A multi-organ nuclei segmentation and classification challenge. IEEE Trans. Med. Imaging *40*, 3413–3423. https://doi.org/10.1109/TMI.2021.3085712.
12. Maishi, N., Annan, D.A., Kikuchi, H., Hida, Y., and Hida, K. (2019). Tumor endothelial heterogeneity in cancer progression. Cancers *11*, 1511. https://doi.org/10.3390/cancers11101511.
13. Diao, J.A., Wang, J.K., Chui, W.F., Mountain, V., Gullapally, S.C., Srinivasan, R., Mitchell, R.N., Glass, B., Hoffman, S., Rao, S.K., et al. (2021). Human-interpretable image features derived from densely mapped cancer pathology slides predict diverse molecular phenotypes. Nat. Commun. *12*, 1613. https://doi.org/10.1038/s41467-021-21896-9.
14. Ing, N., Huang, F., Conley, A., You, S., Ma, Z., Klimov, S., Ohe, C., Yuan, X., Amin, M.B., Figlin, R., et al. (2017). A novel machine learning approach reveals latent vascular phenotypes predictive of renal cancer outcome. Sci. Rep. *7*, 13190. https://doi.org/10.1038/s41598-017-13196-4.
15. Liu, Y., Li, X., Zheng, A., Zhu, X., Liu, S., Hu, M., Luo, Q., Liao, H., Liu, M., He, Y., et al. (2020). Predict Ki-67 positive cells in H&E-stained images using deep learning independently from IHC-stained images. Front. Mol. Biosci. *7*.
16. Jackson, C.R., Sriharan, A., and Vaickus, L.J. (2020). A machine learning algorithm for simulating immunohistochemistry: development of SOX10 virtual IHC and evaluation on primarily melanocytic neoplasms. Mod. Pathol. *33*, 1638–1648. https://doi.org/10.1038/s41379-020-0526-z.
17. Bulten, W., Bándi, P., Hoven, J., Loo, R.v.d., Lotz, J., Weiss, N., Laak, J.v.d., Ginneken, B.v., Hulsbergen-van de Kaa, C., and Litjens, G. (2019). Epithelium segmentation using deep learning in H&E-stained prostate specimens with immunohistochemistry as reference standard. Sci. Rep. *9*, 864. https://doi.org/10.1038/s41598-018-37257-4.
18. Hinton, J.P., Dvorak, K., Roberts, E., French, W.J., Grubbs, J.C., Cress, A.E., Tiwari, H.A., and Nagle, R.B. (2019). A method to reuse archived H&E stained histology slides for a multiplex protein biomarker analysis. Methods Protoc. *2*, E86. https://doi.org/10.3390/mps2040086.
19. Stringer, C., Wang, T., Michaelos, M., and Pachitariu, M. (2021). Cellpose: a generalist algorithm for cellular segmentation. Nat. Methods *18*, 100–106. https://doi.org/10.1038/s41592-020-01018-x.
20. Otsu, N. (1979). A threshold selection method from Gray-level histograms. IEEE Trans. Syst. Man Cybern. *9*, 62–66. https://doi.org/10.1109/TSMC.1979.4310076.
21. The Human Protein Atlas https://www.proteinatlas.org/.
22. Uhlén, M., Fagerberg, L., Hallström, B.M., Lindskog, C., Oksvold, P., Mardinoglu, A., Sivertsson, Å., Kampf, C., Sjöstedt, E., Asplund, A., et al. (2015). Proteomics. Tissue-based map of the human proteome. Science *347*, 1260419. https://doi.org/10.1126/science.1260419.
23. Ross, M.H., and Pawlina, W. (2016). Histology: a text and atlas: with correlated cell and molecular biology, Seventh edition (Wolters Kluwer Health).
24. Campanella, G., Hanna, M.G., Geneslaw, L., Miraflor, A., Werneck Krauss Silva, V., Busam, K.J., Brogi, E., Reuter, V.E., Klimstra, D.S., and Fuchs, T.J. (2019). Clinical-grade computational pathology using weakly supervised deep learning on whole slide images. Nat. Med. *25*, 1301–1309. https://doi.org/10.1038/s41591-019-0508-1.
25. Giraldo, N.A., Sanchez-Salas, R., Peske, J.D., Vano, Y., Becht, E., Petitprez, F., Validire, P., Ingels, A., Cathelineau, X., Fridman, W.H., et al. (2019). The clinical role of the TME in solid cancer. Br. J. Cancer *120*, 45–53. https://doi.org/10.1038/s41416-018-0327-z.
26. German, Y., Vulliard, L., Kamnev, A., Pfajfer, L., Huemer, J., Mautner, A.-K., Rubio, A., Kalinichenko, A., Boztug, K., Ferrand, A., et al. (2021). Morphological profiling of human T and NK lymphocytes by high-content cell imaging. Cell Rep. *36*, 109318. https://doi.org/10.1016/j.celrep.2021.109318.
27. Noguera-Troise, I., Daly, C., Papadopoulos, N.J., Coetzee, S., Boland, P., Gale, N.W., Lin, H.C., Yancopoulos, G.D., and Thurston, G. (2006). Blockade of Dll4 inhibits tumour growth by promoting non-productive angiogenesis. Nature *444*, 1032–1037. https://doi.org/10.1038/nature05355.
28. Hickey, J.W., Tan, Y., Nolan, G.P., and Goltsev, Y. (2021). Strategies for accurate cell type identification in CODEX multiplexed imaging data. Front. Immunol. *12*, 727626.
29. Kuett, L., Catena, R., Özcan, A., Plüss, A., Cancer Grand Challenges IMAXT Consortium, Schraml, P., Moch, H., de Souza, N., and Bodenmiller, B. (2022). Three-dimensional imaging mass cytometry for highly multiplexed molecular and cellular mapping of tissues and the tumor microenvironment. Nat. Cancer *3*, 122–133. https://doi.org/10.1038/s43018-021-00301-w.

CellPress
OPEN ACCESS

30. Kiuru, M., Kriner, M.A., Wong, S., Zhu, G., Terrell, J.R., Li, Q., Hoang, M., Beechem, J., and McPherson, J.D. (2022). High-plex spatial RNA profiling reveals cell type-specific biomarker expression during melanoma development. J. Invest. Dermatol. *142*, 1401–1412.e20. https://doi.org/10.1016/j.jid.2021.06.041.

31. Hickey, J.W., Neumann, E.K., Radtke, A.J., Camarillo, J.M., Beuschel, R.T., Albanese, A., McDonough, E., Hatler, J., Wiblin, A.E., Fisher, J., et al. (2022). Spatial mapping of protein composition and tissue organization: a primer for multiplexed antibody-based imaging. Nat. Methods *19*, 284–295. https://doi.org/10.1038/s41592-021-01316-y.

32. de Haan, K., Zhang, Y., Zuckerman, J.E., Liu, T., Sisk, A.E., Diaz, M.F.P., Jen, K.-Y., Nobori, A., Liou, S., Zhang, S., et al. (2021). Deep learning-based transformation of H&E stained tissues into special stains. Nat. Commun. *12*, 4884. https://doi.org/10.1038/s41467-021-25221-2.

33. Cifci, D., Foersch, S., and Kather, J.N. (2022). Artificial intelligence to identify genetic alterations in conventional histopathology. J. Pathol. *257*, 430–444. https://doi.org/10.1002/path.5898.

34. Vorontsov, E., and Kadoury, S. (2021). Label noise in segmentation networks : mitigation must deal with bias. Preprint at arXiv. https://doi.org/10.48550/arXiv.2107.02189.

35. Kumar, A., and Amid, E. (2021). Constrained instance and class reweighting for robust learning under label noise. Preprint at arXiv. https://doi.org/10.48550/arXiv.2111.05428.

36. Barisoni, L., Lafata, K.J., Hewitt, S.M., Madabhushi, A., and Balis, U.G.J. (2020). Digital pathology and computational image analysis in nephropathology. Nat. Rev. Nephrol. *16*, 669–685. https://doi.org/10.1038/s41581-020-0321-6.

37. Pech-Pacheco, J.L., Cristobal, G., Chamorro-Martinez, J., and Fernandez-Valdivia, J. (2000). Diatom autofocusing in brightfield microscopy: a comparative study. In Proceedings 15th International Conference on Pattern Recognition. ICPR-2000, *3*, pp. 314–317. https://doi.org/10.1109/ICPR.2000.903548.

38. True, L.D. (2008). Quality control in molecular immunohistochemistry. Histochem. Cell Biol. *130*, 473–480. https://doi.org/10.1007/s00418-008-0481-0.

39. Thirion, J.-P. (1998). Image matching as a diffusion process: an analogy with Maxwell's demons. Med. Image Anal. *2*, 243–260. https://doi.org/10.1016/S1361-8415(98)80022-4.

40. Ester, M., Kriegel, H.-P., Sander, J., and Xu, X. (1996). A density-based algorithm for discovering clusters in large spatial databases with noise. In Proceedings of the Second International Conference on Knowledge Discovery and Data Mining KDD'96 (AAAI Press), pp. 226–231.

41. He, K., Zhang, X., Ren, S., and Sun, J. (2015). Deep residual learning for image recognition. Preprint at arXiv. https://doi.org/10.48550/arXiv.1512.03385.

42. Tan, M., and Le, Q.V. (2020). EfficientNet: Rethinking model scaling for convolutional neural networks. Preprint at arXiv. https://doi.org/10.48550/arXiv.1905.11946.

43. Sun, C., Shrivastava, A., Singh, S., and Gupta, A. (2017). Revisiting unreasonable effectiveness of data in deep learning era. Preprint at arXiv. https://doi.org/10.48550/arXiv.1911.04252.

44. Xie, Q., Luong, M.-T., Hovy, E., and Le, Q.V. (2020). Self-training with Noisy Student improves ImageNet classification. Preprint at arXiv. https://doi.org/10.48550/arXiv.1911.04252.

45. Ronneberger, O., Fischer, P., and Brox, T. (2015). U-Net: Convolutional Networks for Biomedical Image Segmentation. Preprint at arXiv. https://doi.org/10.48550/arXiv.1505.04597.

46. Zhou, Z., Siddiquee, M.M.R., Tajbakhsh, N., and Liang, J. (2018). UNet++: A Nested U-Net Architecture for Medical Image Segmentation. Preprint at arXiv. https://doi.org/10.48550/arXiv.1807.10165.

47. Chen, L.-C., Zhu, Y., Papandreou, G., Schroff, F., and Adam, H. (2018). Encoder-Decoder with Atrous Separable Convolution for Semantic Image Segmentation. arXiv. https://doi.org/10.48550/arXiv.1802.02611.

48. Bergstra, J., Bardenet, R., Bengio, Y., and Kégl, B. (2011). Algorithms for Hyper-Parameter Optimization. In Advances in Neural Information Processing Systems (Curran Associates, Inc.).

49. Akiba, T., Sano, S., Yanase, T., Ohta, T., and Koyama, M. (2019). Optuna: A Next-generation Hyperparameter Optimization Framework. Preprint at arXiv. https://doi.org/10.48550/arXiv.1907.10902.

Patterns, Volume *4*

Supplemental information

Restaining-based annotation for cancer histology segmentation to overcome annotation-related limitations among pathologists

Daisuke Komura, Takumi Onoyama, Koki Shinbo, Hiroto Odaka, Minako Hayakawa, Mieko Ochi, Ranny Rahaningrum Herdiantoputri, Haruya Endo, Hiroto Katoh, Tohru Ikeda, Tetsuo Ushiku, and Shumpei Ishikawa

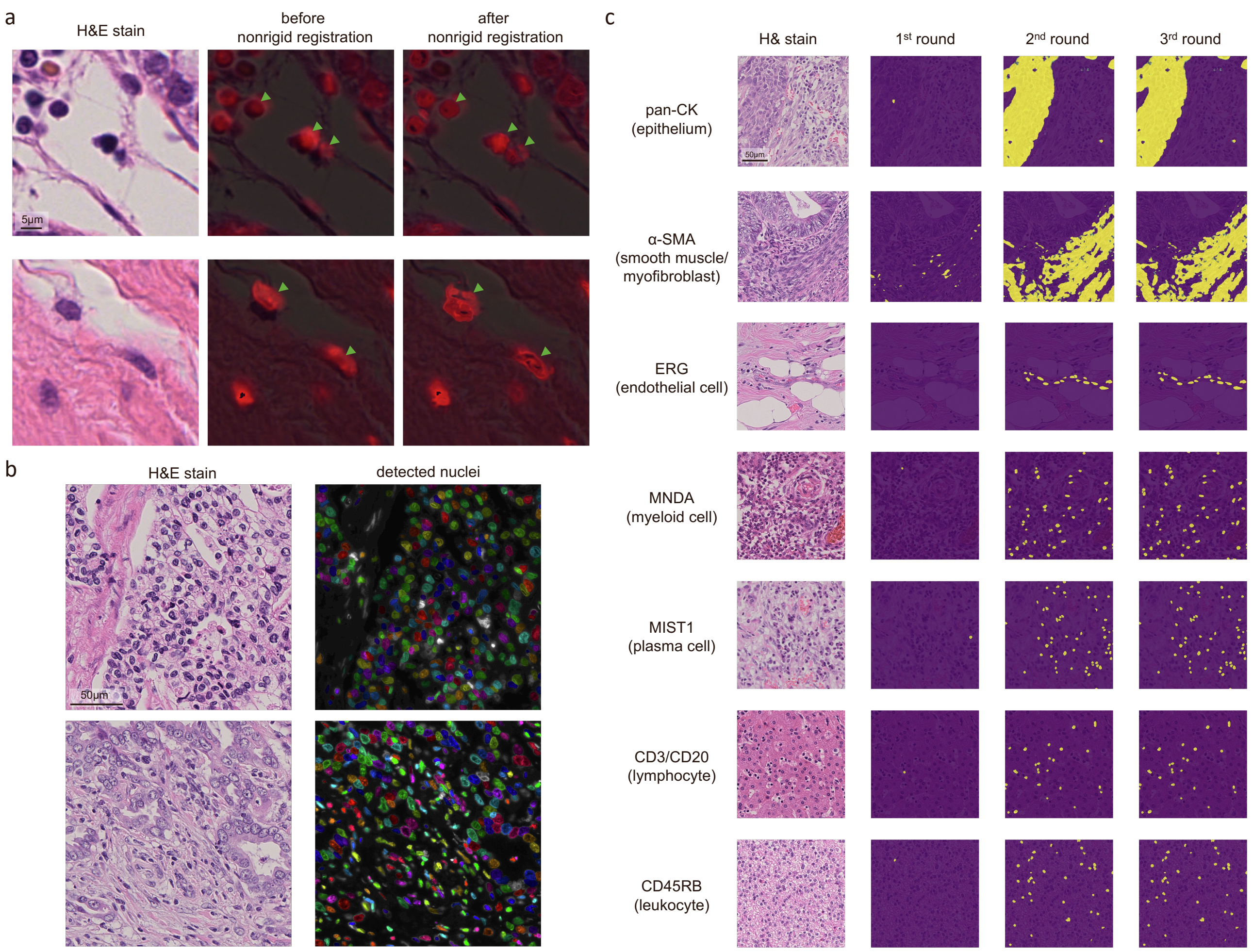

Figure S1. Preprocessing of H&E and the IF slides, related to Figure 1. a, H&E-stained image, along with DAPI channel images before and after nonrigid registration between estimated haematoxylin components in H&E-stained image. H&E images are overlayed in the DAPI channel images. The DAPI component is shown in red, which is converted from blue to improve visibility, in the middle and right images. Arrowheads indicate cells drastically corrected by nonrigid registration. **b,** H&E-stained image and detected nuclei in DAPI component in IF slides. Colour regions represent detected nuclei and grey regions represent nuclei candidates that did not reach the detection threshold. **c,** IF threshold improvement in each phase. The scales of all the image patches in each panel are the same.

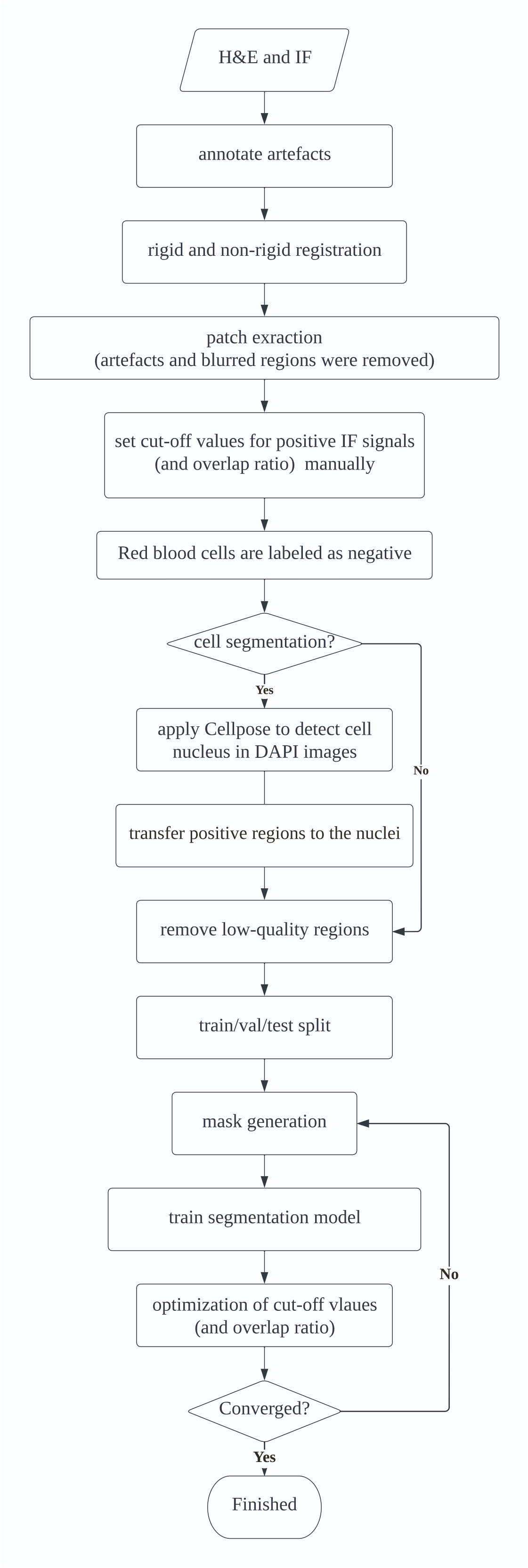

Figure S2. Flow chart of SegPath generation, related to Figure 1.

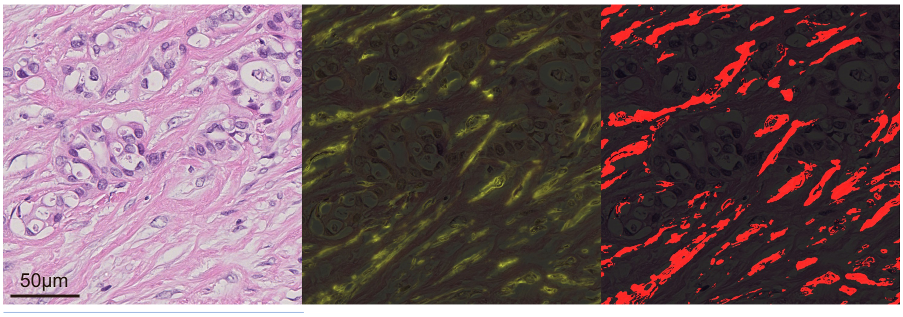

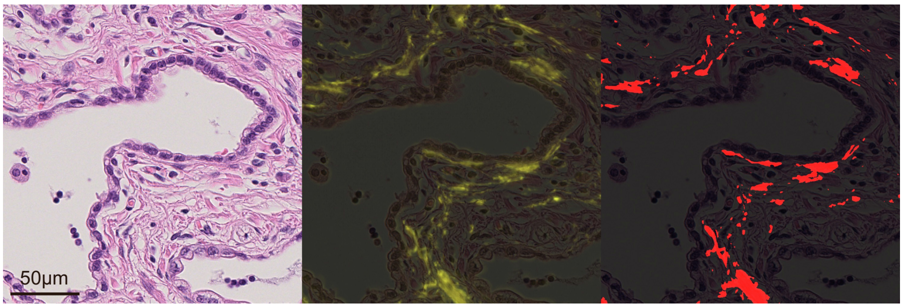

Figure S3. Weak staining of αSMA, related to Figure 3. Each triplet shows an H&E-stained image, the corresponding registered IF image, and the generated mask image (positive regions are indicated in red), from left to right, respectively. The organ was shown above each triplet.

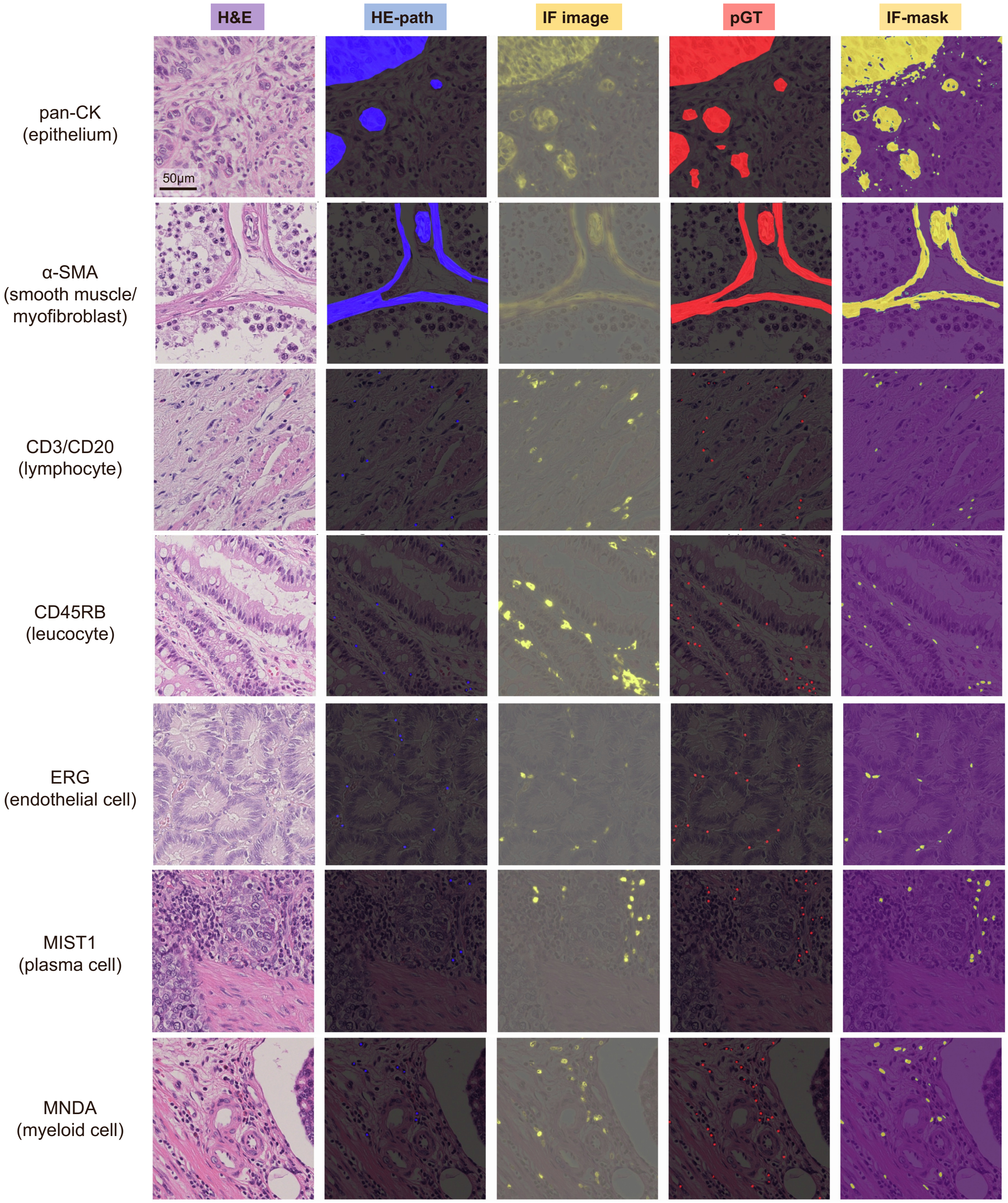

Figure S4. Annotation samples, related to Figure 4. H&E images (first column) and the corresponding annotations for pathologist annotations based on the HE-path images (second column), IF images (third column), HE-path and IF images (fourth column), and IF-mask (fifth column). From the second to fifth columns, the images are overlaid with the corresponding H&E images. The scales of all the image patches are the same.

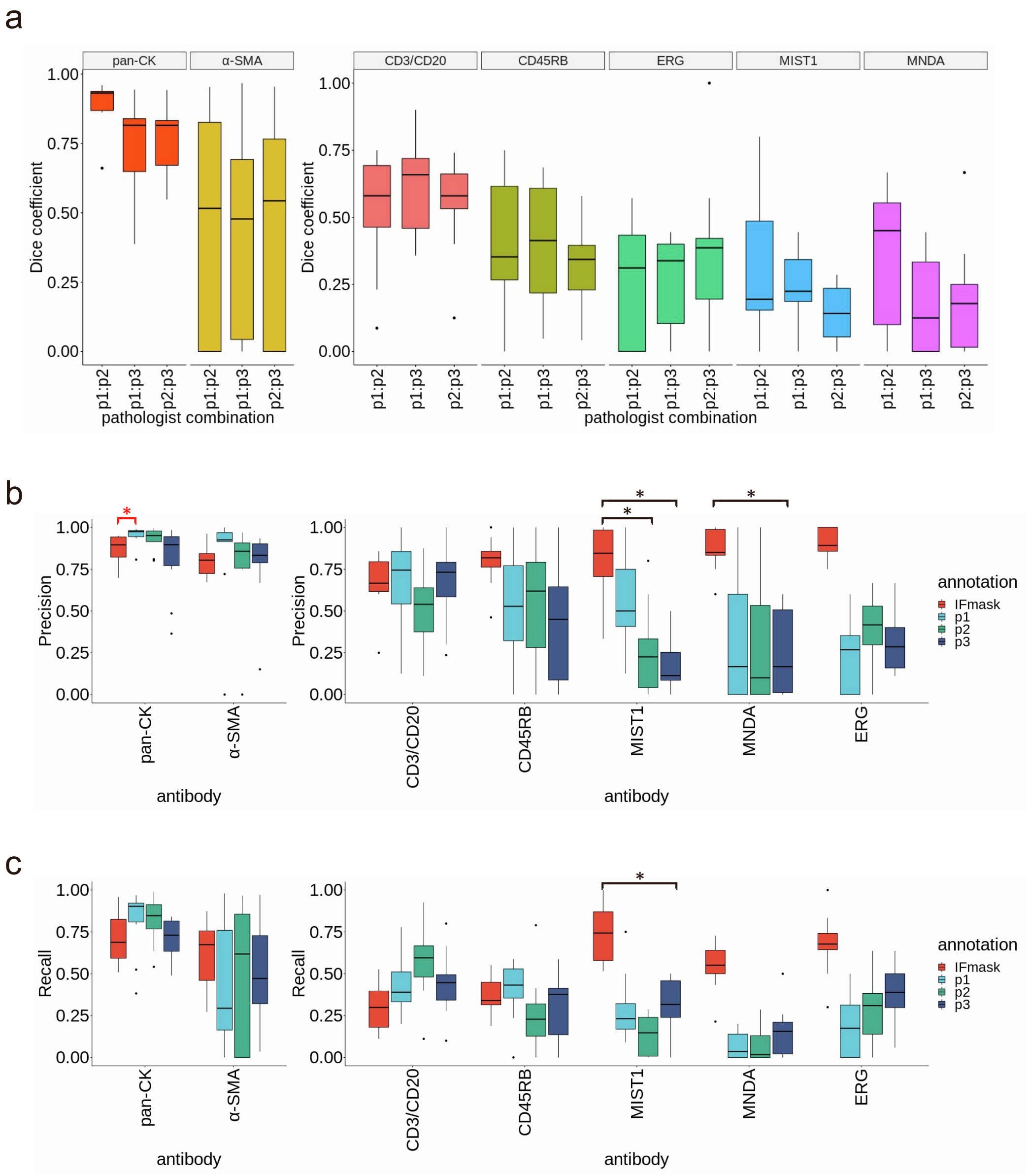

Figure S5. Evaluation of the annotation accuracy of SegPath, related to Figure 4. a, Inter-pathologist concordance of the annotations in terms of Dice coefficients (n = 10 patches of 217.5 × 217.5 μm for each tissue or cell type). Three pathologists annotated the tissues or cells based on the H&E images only. **b, c,** Comparison of the annotation accuracies between pathologists and the automatically generated masks in terms of precision shown in **b** and recall shown in **c**. (n = 10 patches of 217.5 × 217.5 μm for each tissue or cell type). pGT annotations were performed by pathologists who examined both the H&E and corresponding IF images. Regions or cells annotated by at least two of three pathologists were used. *$P < 0.05$.

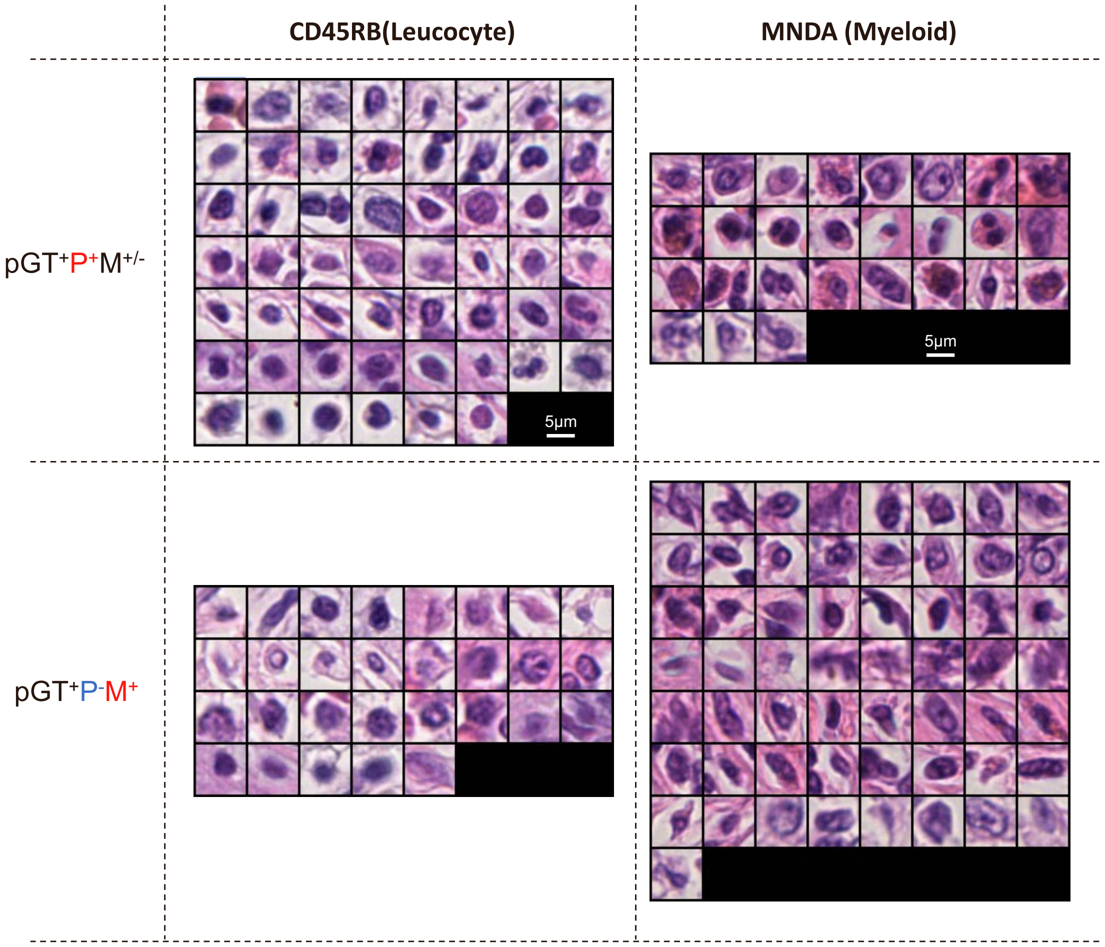

Figure S6. Annotated cell images for leucocytes and myeloid cells, related to Figure 4. pGT cell images annotated by multiple pathologists (pGT^{+}P^{+}M^{+-}) and not identified by multiple pathologists but successfully annotated using the masks (pGT^{+}P^{-}M^{+}). pGT, ground truth; P, HE-path; M, IF-mask.

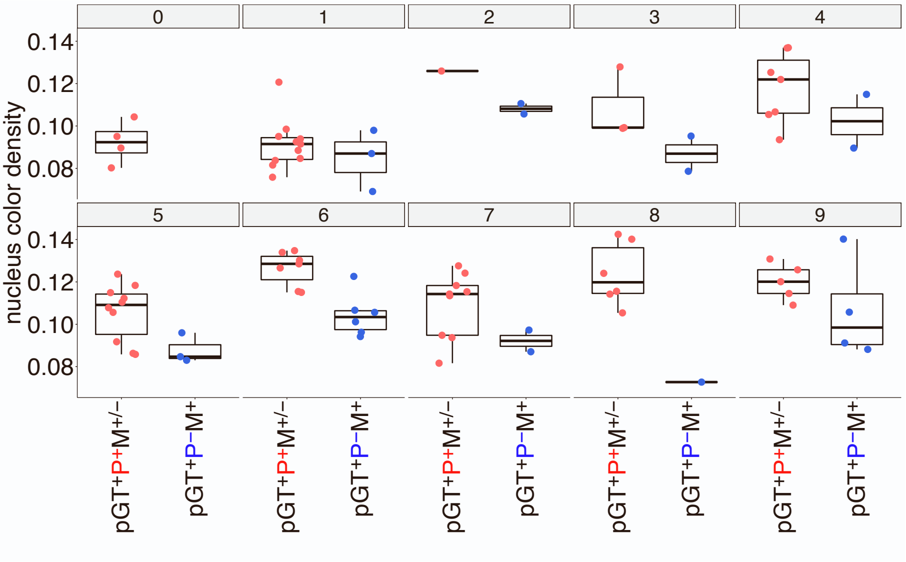

Figure S7. Nucleus haematoxylin intensity of lymphocytes for each patch, related to Figure 4. pGT, ground truth; P, HE-path; M, IF-mask.

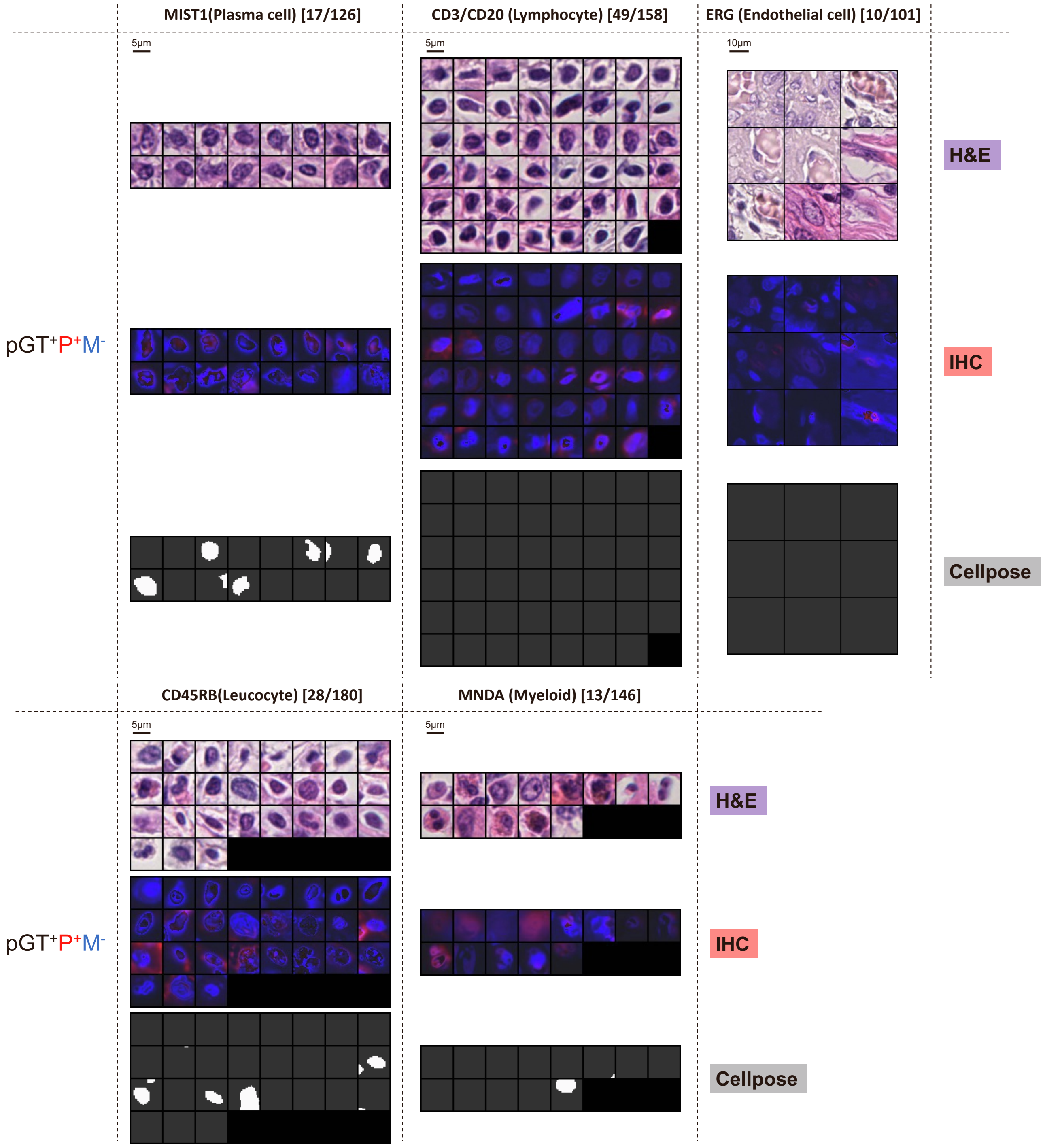

Figure S8. Error analysis of cells correctly identified by pathologists but missed by IF-masks, related to Figure 4. pGT cell images were annotated by multiple pathologists but were not identified by the masks ($pGT^+P^-M^-$). The corresponding IF images of the cell-specific antibodies (red), DAPI nuclear staining (blue), and nuclei detected by Cellpose (white) are shown. The numbers in brackets indicate the number of cells correctly identified by pathologists but missed when using the IF-masks, and the number of pGT cells. pGT, ground truth; P, HE-path; M, IF-mask.

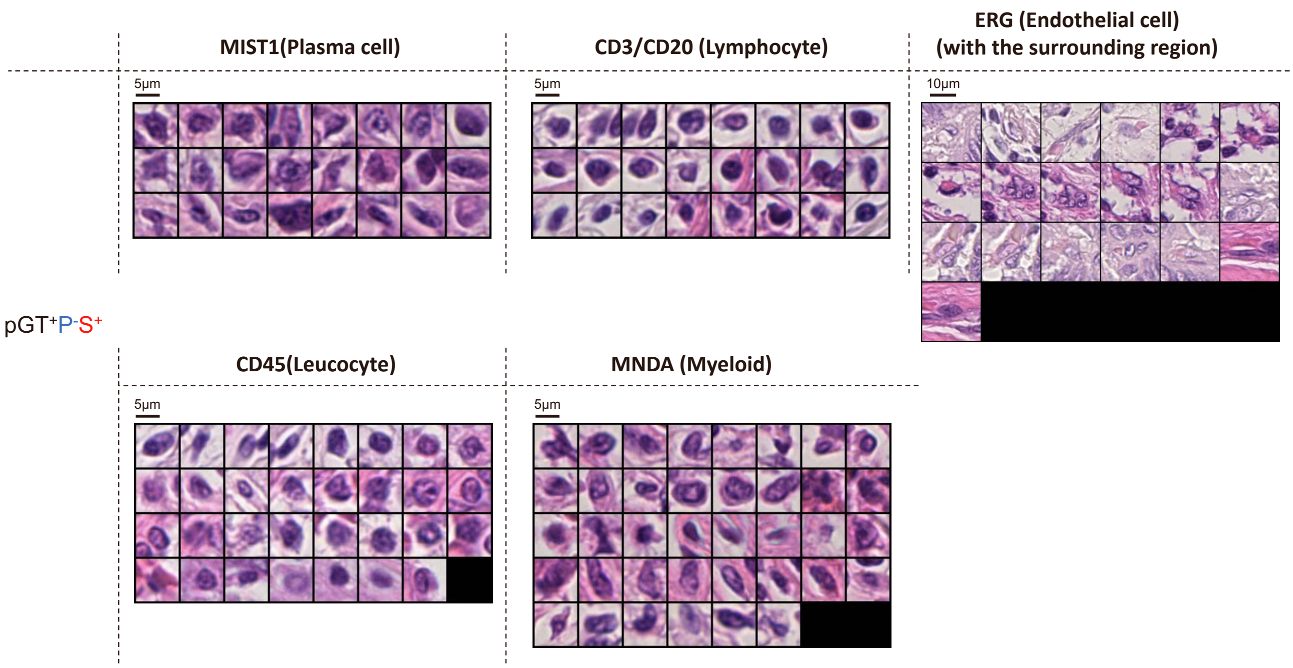

Figure S9. Annotated cell images, related to Figure 6. pGT cell images identified by multiple pathologists but not predicted when using the segmentation models ($pGT^{+}P^{+}S^{-}$). pGT, ground truth; P, HE-path; S, Prediction using the segmentation model. The scales of all the image patches for each cell type are the same.

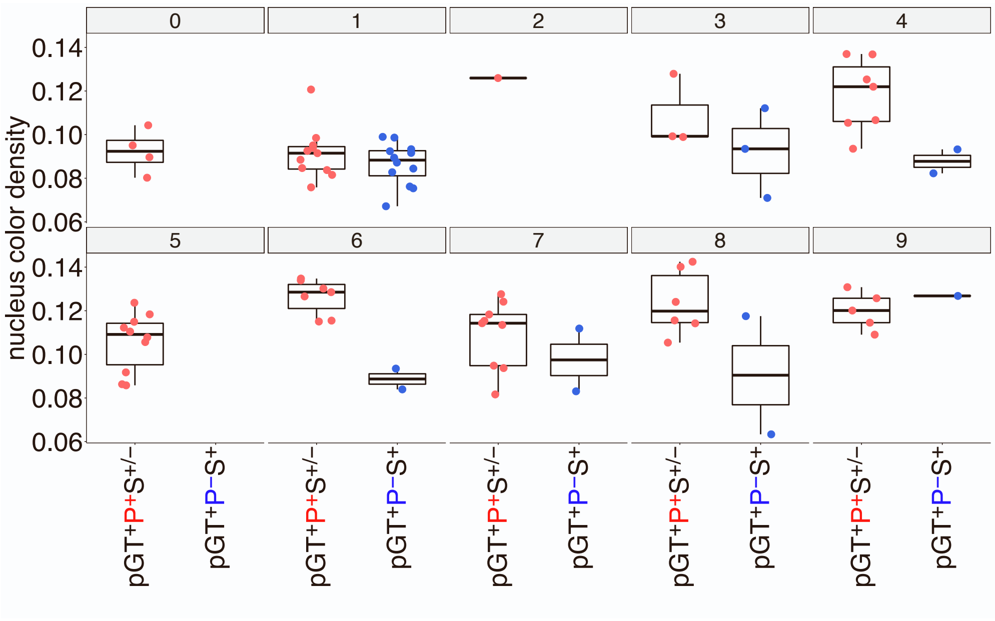

Figure S10. Nucleus haematoxylin intensity of lymphocytes, related to Figure 6. pGT, ground truth; P, HE-path; S, Prediction using the segmentation model.

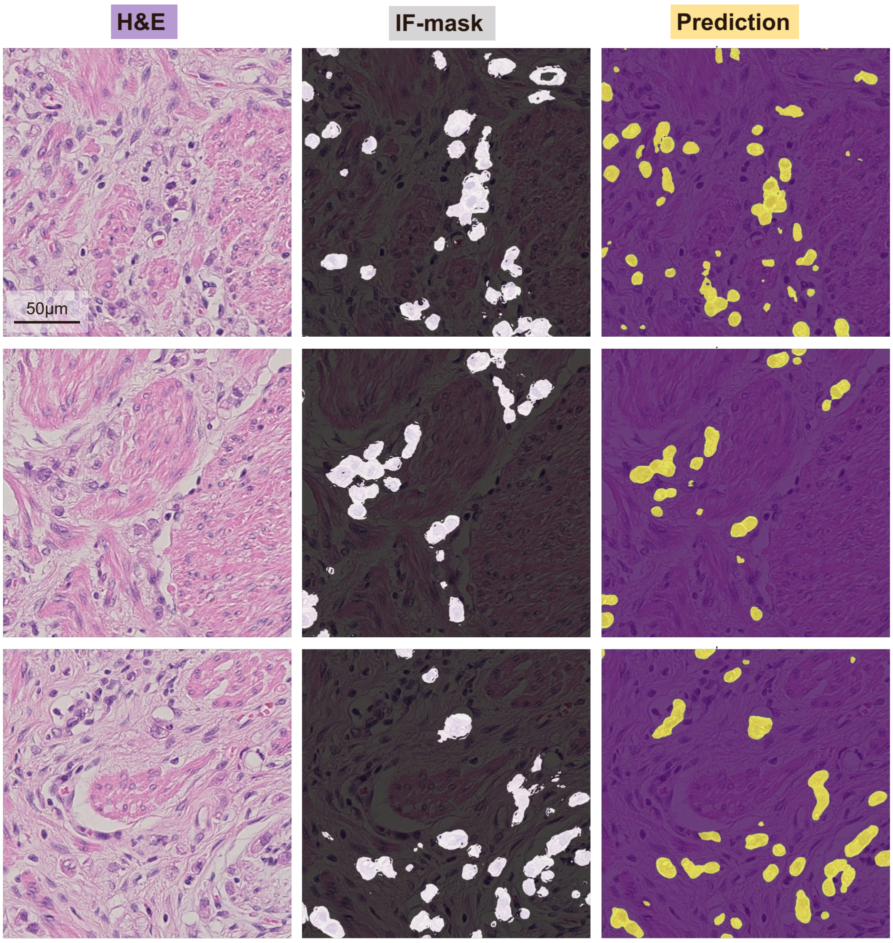

Figure S11. Isolated gastric cancer cells, related to Figure 6. HE-stained images (first columns), IF-masks (second columns), and predicted epithelium regions by the trained segmentation model (third columns) are shown for three different regions. The scales of all the image patches are the same.

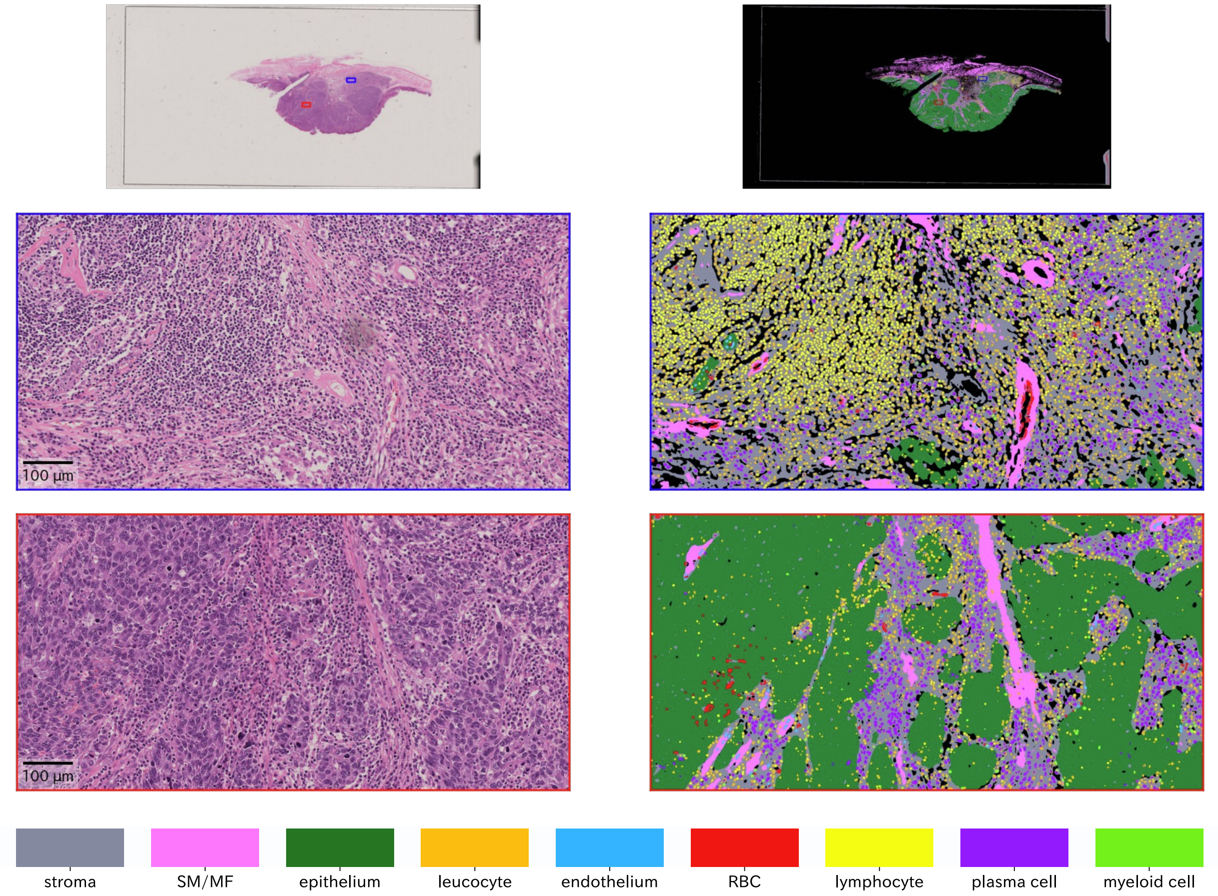

Figure S12. Visualisation of the multi-cell/tissue segmentation results by the trained segmentation models, related to Figure 6. WSI level segmentation of a gastric cancer specimen using the segmentation models. Regions in blue and red rectangles in the top WSI level image are enlarged in the middle and bottom images with a bounding box of the same colour. Different colours represent different cell types as indicated. Grey regions annotated as stroma were tissue regions not detected by any segmentation models. SM, smooth muscle; MF, myofibroblast.

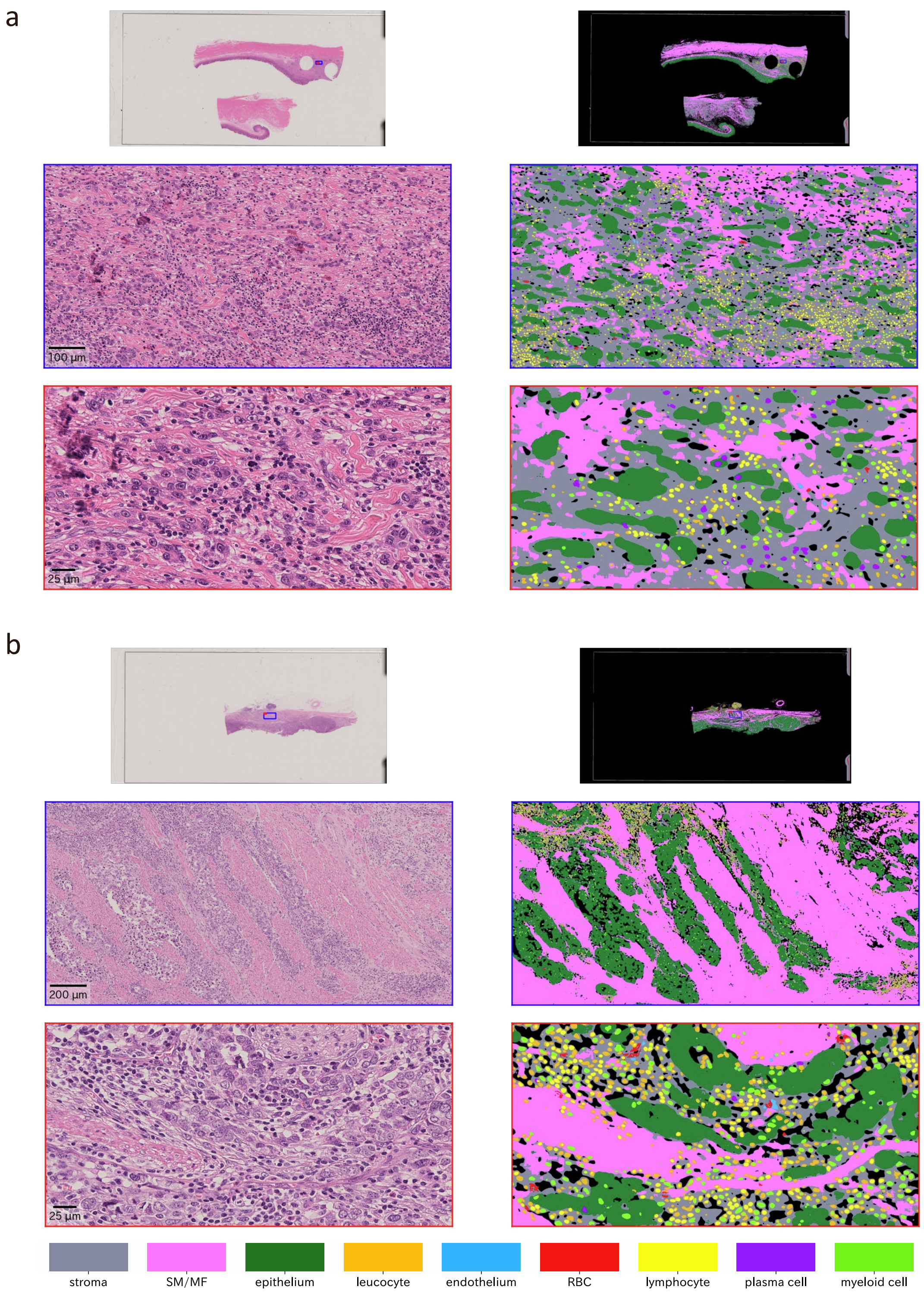

Figure S13. Visualisation of the multi-cell/tissue segmentation results by the trained segmentation models, related to Figure 6. a, b, Gastric cancer cases. SM, smooth muscle; MF, myofibroblast. The regions in the blue and red rectangles in the top WSI level image are enlarged in the middle and bottom images with a bounding box of the same colour.

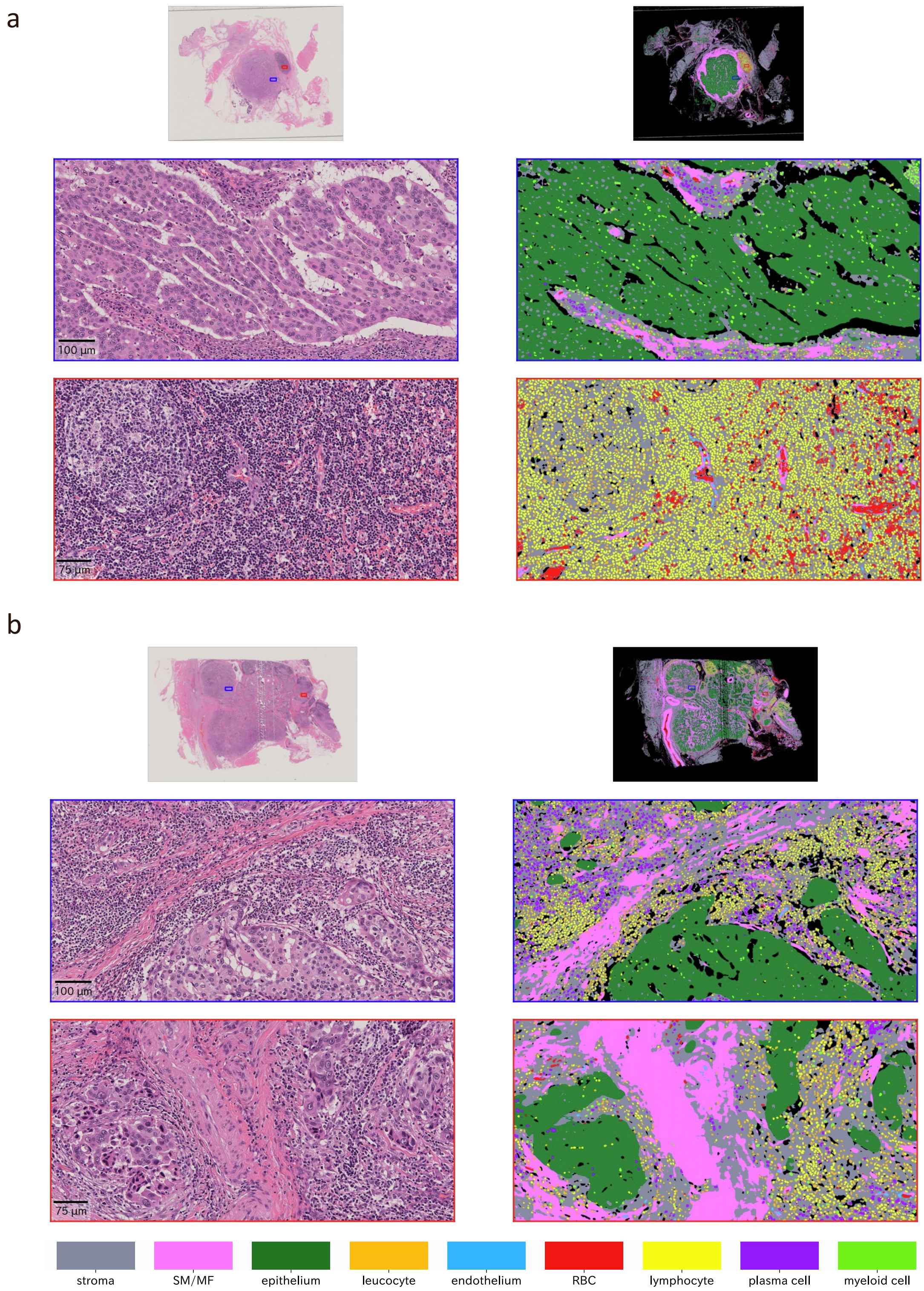

Figure S14. Visualisation of the multi-cell/tissue segmentation results by the trained segmentation models, related to Figure 6. Malignant salivary gland tumours. **a, b,** Salivary duct carcinoma cases. SM, smooth muscle; MF, myofibroblast. The regions in the blue and red rectangles in the top WSI level image are enlarged in the middle and bottom images with a bounding box of the same colour.

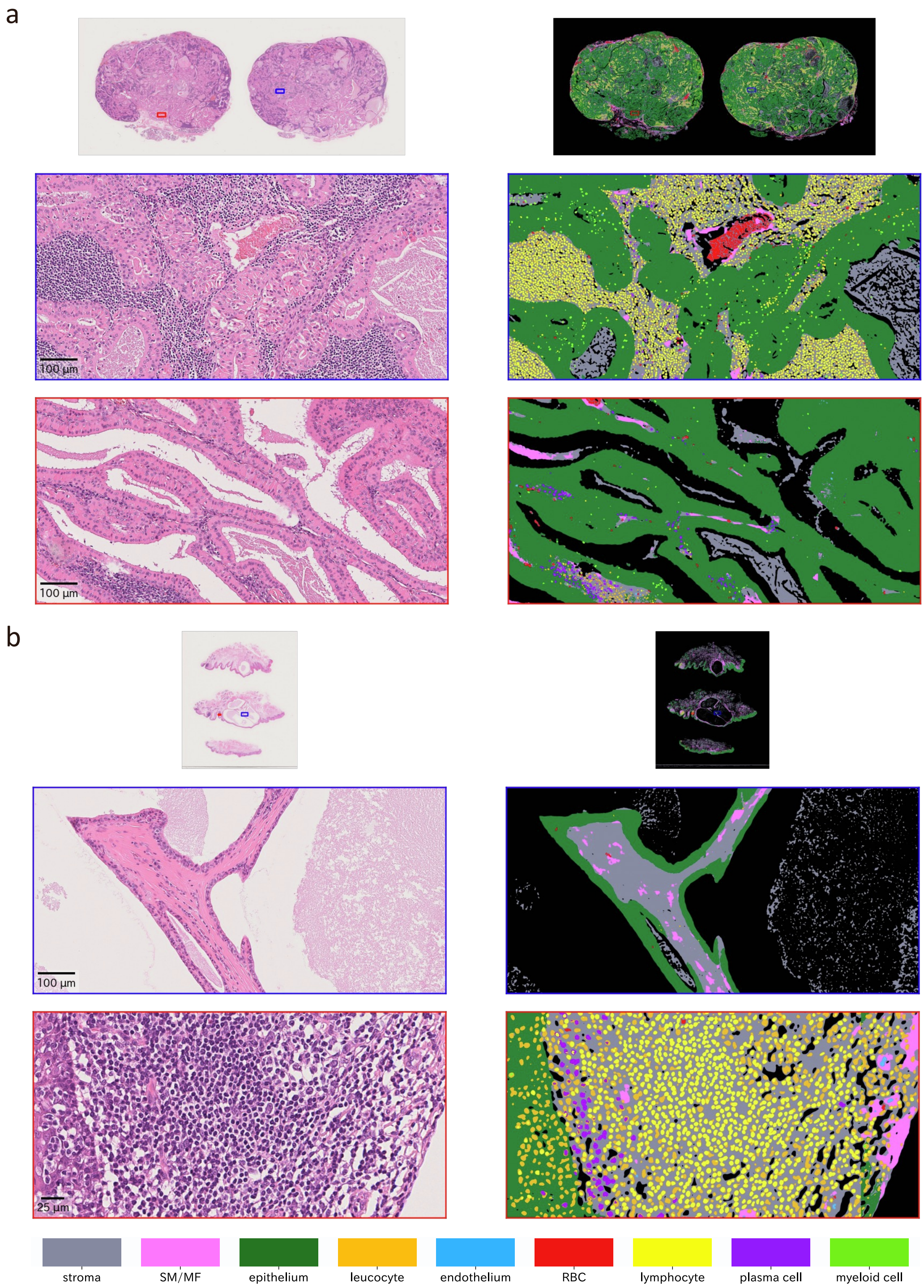

Figure S15. Visualisation of the multi-cell/tissue segmentation results by the trained segmentation models, related to Figure 6. Benign salivary gland tumours. **a,** Warthin's tumour. **b,** Cystadenoma. SM, smooth muscle; MF, myofibroblast. The regions in the blue and red rectangles in the top WSI level image are enlarged in the middle and bottom images with a bounding box of the same colour.

Table S1. Percentage of pixels changed by the threshold determined using the model with the second highest validation dice, related to Methods.

antigen	affected pixel (mean, %)	affected pixel (std, %)	affected pixel (median, %)
pan-CK	0.327	1.347	0
α-SMA	0.21	0.824	0
CD3/CD20	0.167	0.639	0.002
CD45RB	0.283	1.409	0.008
ERG	0.053	0.545	0
MIST1	0.072	0.764	0
MNDA	0.045	0.165	0

Table S4. Ranges of hyperparameters optimized with Optuna for the training of deep neural networks for segmentation, related to Methods.

Hyperparameter	Range	Distribution	Notes
loss function	combo (α*dice+(1-α)*focal loss); dice; binary cross entropy; focal tversky; focal loss; log cosh dice	categorical	
loss parameter	combo (α=[0.1-0.9]) ; focal tversky loss or focal loss (β=[3,8], γ=[0.5,3]); focal loss (β=10, γ=2)	uniform	
learning rate	[1E-4, 1E-2]	log uniform	
stochastic weight averaging	True;False	categorical	
model architeture	DeepLabV3Plus; UnetPlusPlus; Unet	categorical	
model_backbone	timm-efficientnet-b1; timm-efficientnet-b2; timm-efficientnet-b3; resnet18; resnet34; resnet50	categorical	
accumulated gradient	True; False	categorical	
encoder_weights	noisy student; imagnet	categorical	"noisy student" was aplied only when the efficient net was used

Table S6. The number of training patches or cells used in the analysis of sample size effect on the performance, related to Figure 5.

Antigen	Target	Target numbers of patches or cells[#]	unit
pan-CK	epithelium	100/500/1,000/5,000/10,000/15,000/21,912	patch
α-SMA	smooth muscle cell or myofibroblast	100/500/1,000/5,000/10,000/20,000/25,748	patch
CD235a	red blood cell	100/500/1,000/5,000/10,000/15,000/21,595	patch
CD45RB	leucocyte	100/500/1,000/5,000/10,000/50,000/100,000/250,000/500,000/727,503	cell
CD3/CD20	lymphocyte	100/500/1,000/5,000/10,000/50,000/100,000/250,000/329,792	cell
ERG	endothelial cell	100/500/1,000/5,000/10,000/25,000/50,000/56,229	cell
MIST1	plasma cell	100/500/1,000/5,000/10,000/25,000/50,000/72,259	cell
MNDA	myeloid cell	100/500/1,000/5,000/10,000/50,000/100,000/150,000/167,451	cell

The last number is the total number of patches or cells in the training dataset, thus only one test was performed.
